# *In Vivo* Evidence for Lysosome Depletion and Impaired Autophagic Clearance in Hereditary Spastic Paraplegia Type SPG11

**DOI:** 10.1371/journal.pgen.1005454

**Published:** 2015-08-18

**Authors:** Rita-Eva Varga, Mukhran Khundadze, Markus Damme, Sandor Nietzsche, Birgit Hoffmann, Tobias Stauber, Nicole Koch, J. Christopher Hennings, Patricia Franzka, Antje K. Huebner, Michael M. Kessels, Christoph Biskup, Thomas J. Jentsch, Britta Qualmann, Thomas Braulke, Ingo Kurth, Christian Beetz, Christian A. Hübner

**Affiliations:** 1 Institute of Human Genetics, Jena University Hospital, Friedrich-Schiller-University Jena, Jena, Germany; 2 Institute of Clinical Chemistry, Jena University Hospital, Friedrich-Schiller-University Jena, Jena, Germany; 3 Biochemical Institute, University of Kiel, Kiel, Germany; 4 Electron Microscopy Center, Jena University Hospital, Friedrich-Schiller-University Jena, Jena, Germany; 5 Biomolecular Photonics Group, Jena University Hospital, Friedrich-Schiller-University Jena, Jena, Germany; 6 Leibniz-Institut für Molekulare Pharmakologie (FMP) und Max-Delbrück Centrum für Molekulare Medizin (MDC), Berlin, Germany; 7 Institute of Biochemistry I, Jena University Hospital, Friedrich-Schiller-University Jena, Jena, Germany; 8 Department of Biochemistry, Children’s Hospital, University Medical Center Hamburg-Eppendorf, Hamburg, Germany; The Jackson Laboratory, UNITED STATES

## Abstract

Hereditary spastic paraplegia (HSP) is characterized by a dying back degeneration of corticospinal axons which leads to progressive weakness and spasticity of the legs. SPG11 is the most common autosomal-recessive form of HSPs and is caused by mutations in *SPG11*. A recent *in vitro* study suggested that Spatacsin, the respective gene product, is needed for the recycling of lysosomes from autolysosomes, a process known as autophagic lysosome reformation. The relevance of this observation for hereditary spastic paraplegia, however, has remained unclear. Here, we report that disruption of Spatacsin in mice indeed causes hereditary spastic paraplegia-like phenotypes with loss of cortical neurons and Purkinje cells. Degenerating neurons accumulate autofluorescent material, which stains for the lysosomal protein Lamp1 and for p62, a marker of substrate destined to be degraded by autophagy, and hence appears to be related to autolysosomes. Supporting a more generalized defect of autophagy, levels of lipidated LC3 are increased in Spatacsin knockout mouse embryonic fibrobasts (MEFs). Though distinct parameters of lysosomal function like processing of cathepsin D and lysosomal pH are preserved, lysosome numbers are reduced in knockout MEFs and the recovery of lysosomes during sustained starvation impaired consistent with a defect of autophagic lysosome reformation. Because lysosomes are reduced in cortical neurons and Purkinje cells *in vivo*, we propose that the decreased number of lysosomes available for fusion with autophagosomes impairs autolysosomal clearance, results in the accumulation of undegraded material and finally causes death of particularly sensitive neurons like cortical motoneurons and Purkinje cells in knockout mice.

## Author Summary

Autophagy is a degradative pathway for the removal and subsequent recycling of dysfunctional intracellular components. The material destined for degradation is initially enclosed by a double membrane, the autophagosome. In autolysosomes, which result from fusion of autophagosomes with lysosomes, the material is finally broken down. Recent *in vitro* data suggested that the protein Spatacsin plays a pivotal role in the regeneration of lysosomes from autolysosomes. Spatacsin is encoded by *SPG11*, the most common gene mutated in autosomal recessive hereditary spastic paraplegia. Here we show that mice devoid of Spatacsin develop symptoms consistent with spastic paraplegia and progressively loose cortical motoneurons and Purkinje cells. In these mice degenerating neurons have a reduced number of lysosomes available for fusion with autophagosomes and consequently accumulate autolysosome-derived material over time. In the long term this causes death of particularly sensitive neurons like cortical motoneurons and Purkinje cells.

## Introduction

Hereditary spastic paraplegias (HSPs) are a group of movement disorders characterized by length-dependent degeneration of upper motoneuron axons resulting in leg weakness and spasticity [1]. More than 70 genetically distinct forms (SPG1-SPG72) are currently recognized [2]. SPG11 represents a complicated form of HSP with cognitive decline, thinning of the corpus callosum, white matter lesions and cerebellar signs among other symptoms very similar to SPG15 [3]. While SPG11 is caused by *SPG11* mutations [4], mutations in *SPG15/ZFYVE26* underlie SPG15 [5]. Suggesting that SPG11 and SPG15 are pathophysiologically linked, the protein products of both *SPG11* and *SPG15*, Spatacsin and Spastizin respectively, associate with the adaptor protein complex 5 (AP-5), which belongs to a group of tetrameric protein complexes involved in vesicular transport [6–8]. Interestingly, mutations in *AP5Z1* encoding the ζ-subunit of AP-5 underlie SPG48 [6], which shares several clinical features with SPG11 and SPG15 [3].

The subcellular localization of the proteins and their suggested respective functions are quite controversial. DNA repair [6], cell division [9], autophagy [10], axon outgrowth [11], and endolysosomal trafficking have been proposed [12,13]. The latter was suggested because knockdown of individual AP-5 subunits in HeLa cells caused the cation-independent mannose 6-phosphate receptor to become trapped in clusters of early endosomes [12]. Also pointing to this direction degenerating neurons in a recent Spastizin knockout mouse model accumulated autofluorescent material in Lamp1-positive vesicular structures [13] and fibroblasts from both SPG11 and SPG15 patients displayed an enlarged Lamp1-positive compartment [14]. Because autophagosome numbers were increased in fibroblasts of SPG15 patients and in knockdown studies with primary mouse neurons, it was further proposed that the fusion of autophagosomes with lysosomes is impaired [10]. This concept has recently been challenged by *in vitro* studies on HeLa cells showing that Spatacsin and Spastizin are essential for the regeneration of lysosomes from autolysosomes, a process known as autophagic lysosome reformation (ALR) [15], which has so far only been observed *in vitro* [16]. According to this model impaired ALR is expected to lead to exhaustion of lysosomes available for fusion of autophagosomes and accumulation of autolysosomes.

We here provide data that neurons in Spatacsin knockout mice accumulate abnormal autolysosomes and autolysosome-related autofluorescent material. Autolysosomes also accumulate in knockout mouse embryonic fibroblasts (MEFs), while their lysosome numbers are decreased. Upon starvation lysosomes are depleted in MEFs of both genotypes, but only recover in wild-type during prolonged starvation in accordance with a defect of the regeneration of lysosomes from autolysosomes. Consistently, lysosomes are reduced in knockout Purkinje cells and cortical motoneurons, even before accumulation of autofluorescent material and overt neurodegeneration. The loss of particularly susceptible neurons like cortical motoneurons and Purkinje cells finally causes the complex neurological phenotype of Spatacsin knockout mice.

## Results

### Generation of Spatacsin knockout mice

**Fig 1:**
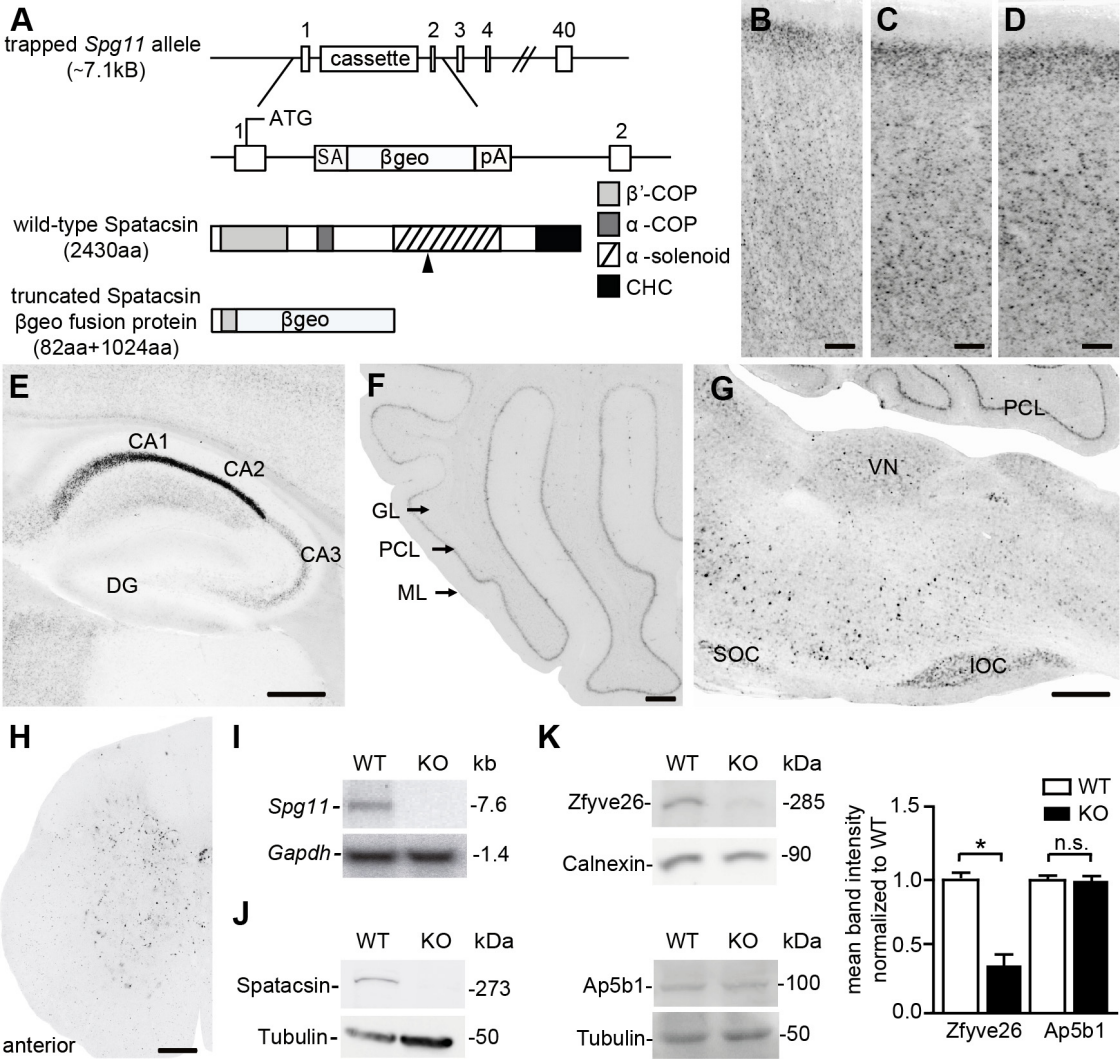
Homozygous trapped mice represent Spatacsin knockout mice. **(A)** Partial genomic structure of the targeted *Spg11* locus and the predicted mutant fusion protein as compared to wild-type Spatacsin; rectangles: exons, SA: splice acceptor, βgeo: β-galactosidase and neomycin fusion cassette, pA: polyadenylation site. The black arrowhead indicates the position of the epitope for antibody generation. CHC: clathrin heavy chain. **(B-H)** LacZ stainings of sections of the cortex (B-D), hippocampus (E), cerebellum (F), brain stem (G), and spinal cord (H) from 2-month-old heterozygous trapped mice shows that *Spg11* expression follows a neuronal pattern. DG: dentate gyrus; GL: granular layer, PCL: Purkinje cell layer, ML: molecular layer; SOC: superior olivary complex, IOC: inferior olivary complex. Scale bars: 100 μm. **(I)** Northern blot analysis of total brain RNA from wild-type (WT) mice shows a 7.6 kb WT-transcript which is absent in RNA isolated from homozygous trapped mice (KO). *Gapdh* served as a loading control. **(J)** Western Blot analysis with an affinity-purified monoclonal antibody directed against the deleted part of the Spatacsin protein (the position of the epitope is indicated in (A) by an arrowhead) detects a band of the predicted size in WT brain lysates, which is absent in brain lysates of homozygous trapped mice. Tubulin served as a loading control. (**K**) While Zfyve26 levels are diminished in brain lysates of Spatacsin KO mice, levels of the beta subunit of the AP-5 complex (Ap5b1) are not changed (n = 3; Student’s t-test: * indicates p<0.05; n.s.: not significant).

To model SPG11 *in vivo*, we injected cells of the ES cell clone EUCE0085_F05 from the European conditional mouse mutagenesis program (EUCOMM) into donor blastocysts. The resulting chimeric mice were mated with C57Bl6 wild-type (WT) mice to obtain mice with a heterozygous trapped locus. Because the gene-trap cassette is integrated into intron 1 of *Spg11* (Fig 1A), the targeted locus is predicted to encode a cytoplasmic fusion protein of the 82 N-terminal amino acids of Spatacsin with βgeo under the control of the endogenous *Spg11* promoter, while the following part of Spatacsin is lost.

The β-galactosidase activity of βgeo allowed us to assess the expression of *Spg11* by LacZ staining of tissue sections of heterozygous trapped mice, which supports a broad expression pattern including cortex and hippocampus (Fig 1B–1E), cerebellum (Fig 1F), neurons of the brain stem (Fig 1G), and the spinal cord (Fig 1H). To get more information on the expression of *Spg11* in different types of neurons we co-stained tissue sections for β-galactosidase and various marker proteins including NeuN, a broad neuronal marker, Ctip2, which preferentially labels layer V neurons [17], and parvalbumin, which is expressed in a subset of interneurons. From these co-stainings it appears that *Spg11* is broadly expressed in different types of neurons including principal cells and inhibitory neurons (S1 Fig).

From matings of heterozygous trapped mice we obtained homozygous targeted offspring in the expected Mendelian ratio. While Northern analysis with a probe corresponding to part of exon 30 of *Spg11* detected transcripts of the expected size in RNA isolated from WT brains, the transcript was absent in RNA isolated from homozygous trapped mice (Fig 1I). To detect the Spatacsin protein we generated monoclonal antibodies directed against an epitope within the α-solenoid domain of Spatacsin (Fig 1A) and affinity-purified the resulting antiserum. Confirming its specificity, the antibody detected a polypeptide of the predicted size of Spatacsin of 273 kDa in brain lysates from WT mice, which was absent from protein lysates isolated from brains of homozygous trapped mice (Fig 1J). Though the antibody was suited for Western blot analysis, it did not detect endogenous Spatacsin in immunostainings.

Because it was shown that Spatacsin co-precipitates with Zfyve26 and subunits of the AP-5 complex [6], we assessed whether Zfyve26 levels are changed in brain lysates of homozygous trapped mice. Indeed, Zfyve26 levels were reduced. In contrast levels of the β-subunit of the AP-5 complex (Ap5b1) were not changed (Fig 1K, though it has been reported that siRNA mediated knockdown of Spatacsin in HeLa cells caused a decrease of levels for the μ5-subunit of the AP-5 complex [12]. Decreased levels AP5B1 were also reported for fibroblasts isolated from SPG11 patients [15].

Our results are consistent with the assumption that the trapped *Spg11* locus corresponds to a null allele and that mice homozygous for the trapped allele represent Spatacsin knockout (KO) mice. Further on, homozygous trapped mice are therefore also referred to as Spatacsin KO mice.

### Spatacsin knockout mice develop a progressive gait disorder with ataxia

**Fig 2:**
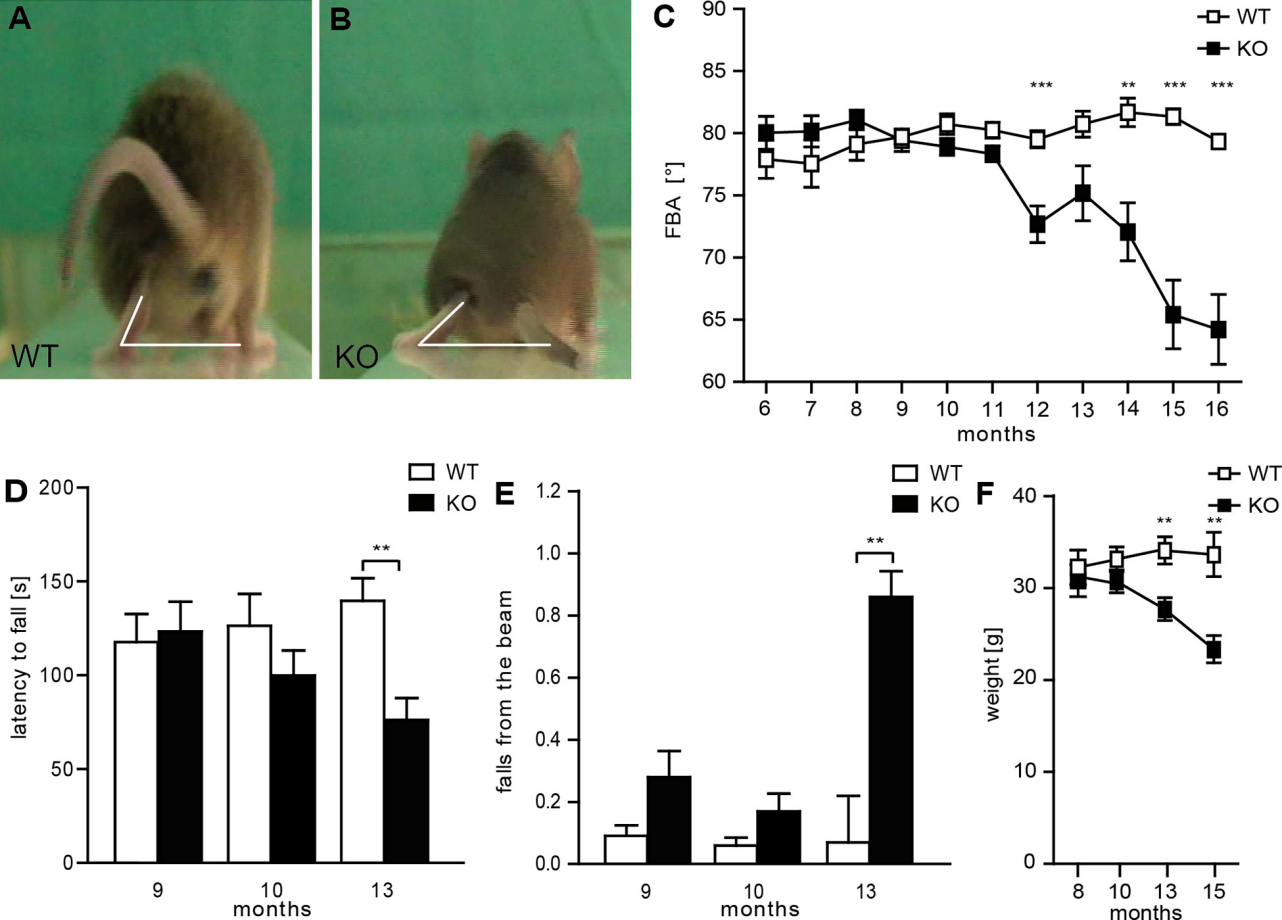
Disruption of Spatacsin in mice results in a severe spastic and ataxic gait disorder. **(A-B)** Representative single video frames of a WT (A) and KO (B) mouse 14 months of age walking on a beam at the moment when the toe of the hind limb is taken off. The foot-base-angle (FBA) is indicated by white lines. **(C)** The FBA decreases over time in KO but not in WT mice (n = 20; 2-way-ANOVA: p<0.0001; Bonferroni post-hoc analysis: *** indicates p<0.001 and ** p<0.01). **(D)** The latency to fall off an accelerating rotating rod decreases over time in KO mice (n = 20; 2-way-ANOVA: p = 0.05; Bonferroni post-hoc analysis: ** indicates p<0.01). **(E)** Compared to control mice aged KO mice fall off the beam more frequently (n = 20; 2-way-ANOVA: p = 0.0157; Bonferroni post-hoc analysis: ** indicates p<0.01). Error bars represent SEM. **(F)** Spatacsin KO mice loose body weight starting around 12 months of age (n = 20; 2-way-ANOVA: p<0.0001; Bonferroni post-hoc analysis: *** indicates p<0.001 and ** p<0.01).

Spatacsin KO mice younger than 12 months of age did not show any obvious motor phenotype compared to WT littermates. Subsequently, KO mice developed a progressive gait disorder. For quantification of the motor phenotype we measured the foot-base-angle (FBA) at toe-off positions of the hind-paws, which decreased with age in KO mice (Fig 2A–2C). Moreover, the latency of KO mice to fall off an accelerating rotating rod decreased in aged mice (Fig 2D). Further suggesting a motor coordination defect the number of falls in the beam walking test was significantly increased at around 13 months of age (Fig 2E). Around this age the body weight of KO mice decreased, consistent with a deterioration of the overall health status of KO mice (Fig 2F). Thus, Spatacsin KO mice show a progressive worsening of motor performance compatible with complex HSP.

### Cortical motoneurons and Purkinje cells are progressively lost in Spatacsin knockout mice

**Fig 3:**
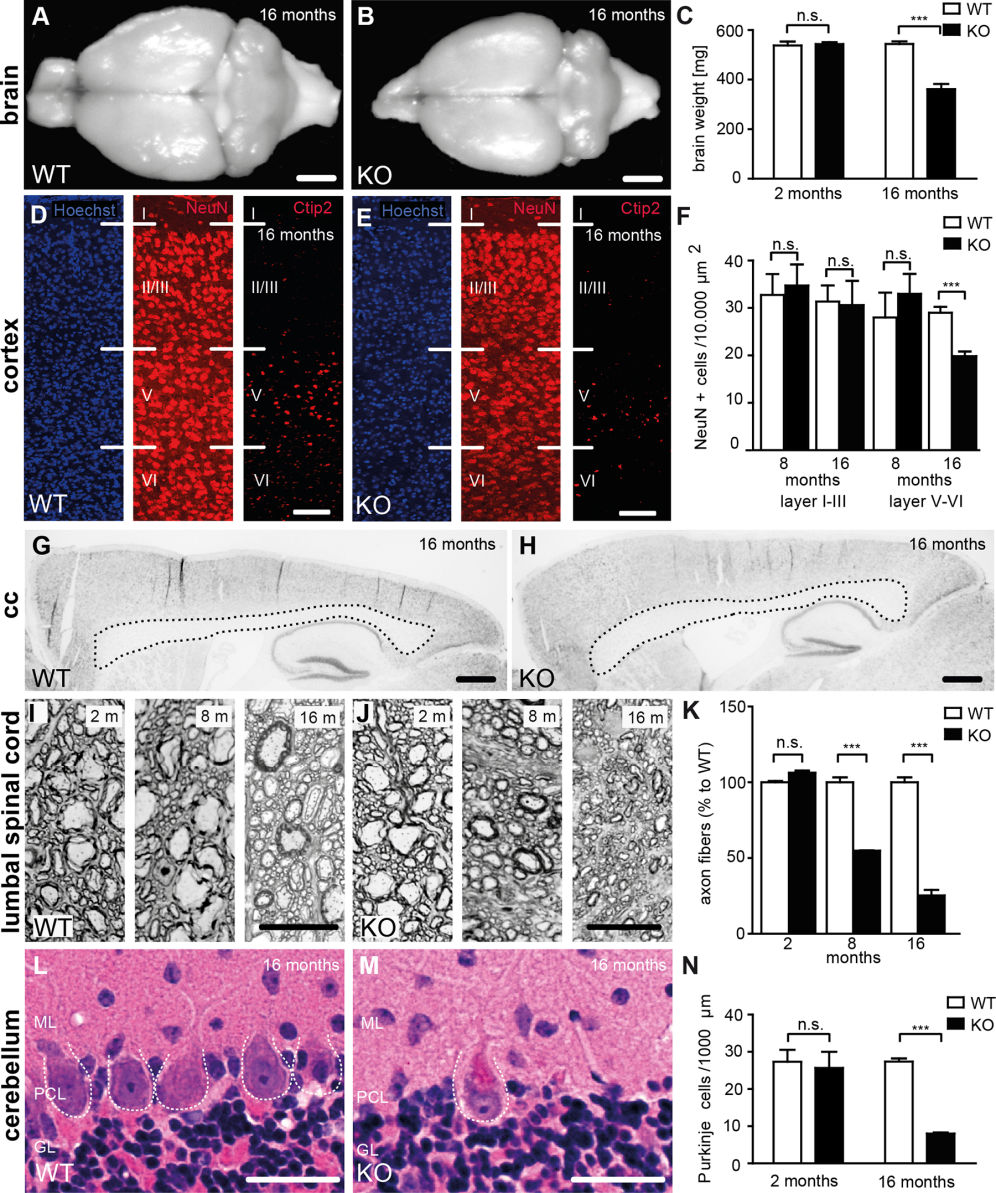
Loss of Spatacsin causes severe neuron loss in the motor cortex and the cerebellum. **(A-B)** Brain size is reduced in 16-month-old KO compared to WT mice. Scale bars: 2 mm. **(C)** Progressive reduction of brain weight in KO mice (n = 3; Student’s t-test: *** indicates p<0.001). **(D-F)** Neuron loss in the motor cortex of 16-month-old KO mice. Hoechst-33258 (blue; nuclei), NeuN (red; neuronal marker), and Ctip2 (red; marker for layer V neurons) staining of the motor cortex at 16 months of age from WT (D) and KO (E) mice. Individual cortical layers are labeled (I–VI). Scale bars: 100 μm. Quantification of NeuN-positive cells reveals a depletion of neurons from layers V-VI but not layers I-III of the motor cortex at 16 months of age in the KO (n = 3; Student’s t-test: *** indicates p<0.001). **(G, H)** Nissl stainings of sagittal brain sections do not support a thinning of the corpus callosum of 16-month-old KO mice. Scale bars: 500 μm (**I-K**) Semithin sections of the lumbar corticospinal tract show a loss of large diameter axons in 8- and 16-month-old KO mice (n = 3; Student’s t-test: *** indicates p<0.001). Scale bars: 20 μm. **(L-N)** Purkinje-cell loss in aged KO (M) but not WT (L) mice. ML: Molecular layer; PCL: Purkinje cell layer; GL: granular layer. The somata of Purkinje cells is indicated by a dotted line. (N) Quantification of Purkinje cells in hematoxylin-eosin stained cerebellum sections indicates a severe loss of Purkinje cells in aged KO mice (n = 3; Student’s t-test: *** indicates p<0.001). Scale bars: 40 μm.

Suggesting a systemic neurodegenerative disorder the brain size (Fig 3A and 3B) and weight (Fig 3C) did not differ around 2 months of age but was reduced in 16-month-old KO mice. Quantification of NeuN-positive neurons of the motor cortex revealed a loss of large projection neurons in cortical layers V to VI at 16 but not at 8 months of age (Fig 3D–3F). This was further confirmed by staining with Ctip2 (Fig 3D and 3E). Neuron numbers in layers I-III, where most of the commissural neurons reside [18], were unchanged, which is consistent with the intact corpus callosum in aged Spatacsin KO mice (Fig 3G and 3H).

Large diameter axon fibers were reduced by roughly 50% at 8 and by roughly 75% at 16 months of age in the lumbar corticospinal tract (L4, Fig 3I–3K). In the cervical corticospinal tract it was reduced by 56% at 16 months of age (n = 3; Student’s t-test: p<0.001). In contrast to previous results from zebra fish [19] the overall structure of motor-endplates was not altered in KO mice (S2 Fig).

Purkinje cells were drastically reduced (Fig 3L–3N), while numbers of pyramidal cells in the hippocampus and spinal cord motoneurons were not changed in 16-month-old KO mice (S3 Fig).

**Fig 4:**
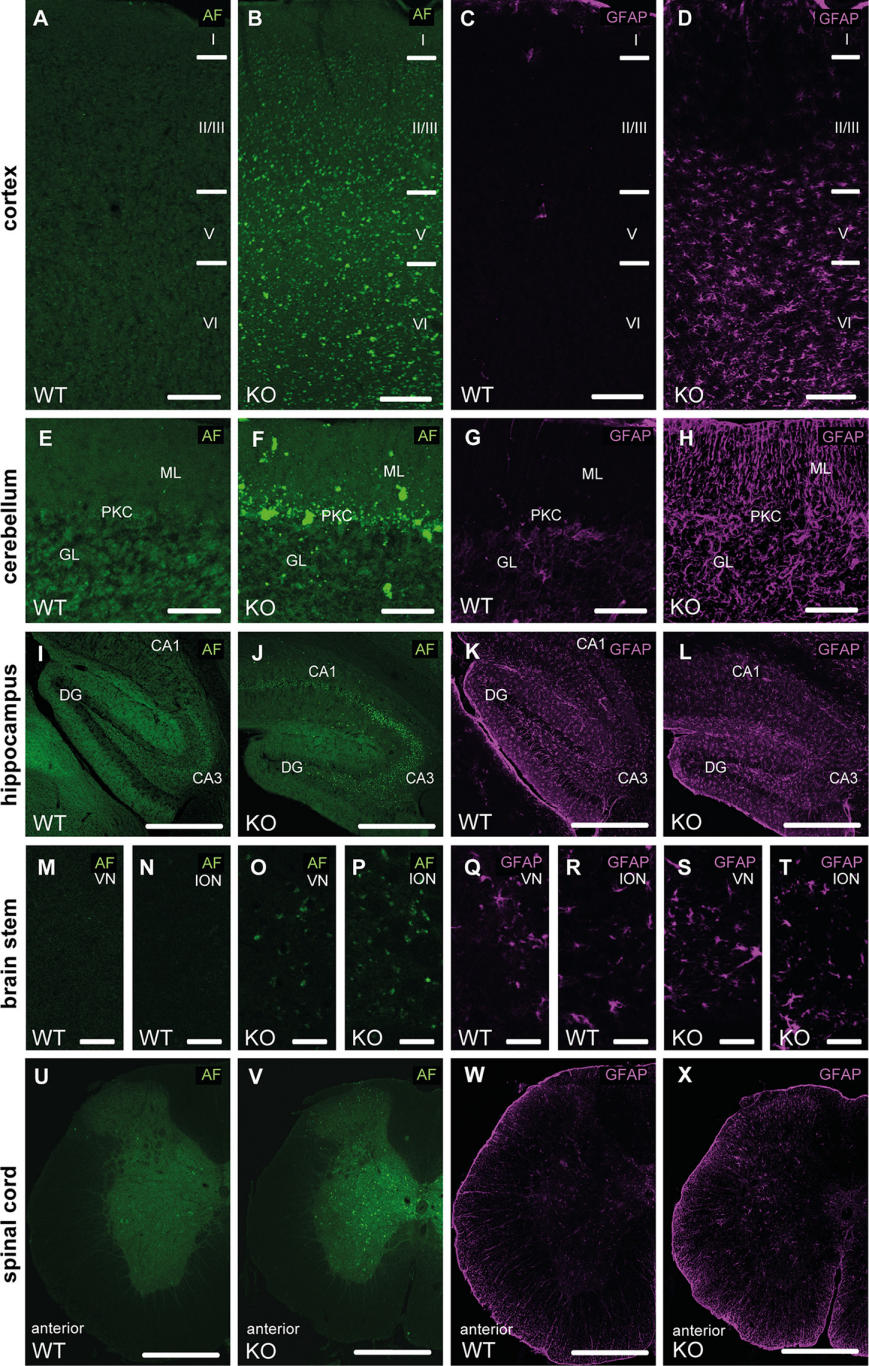
Autofluorescence and GFAP immunoreactivity in different regions of the central nervous system. **(A-D)** At 16 months of age we observed an accumulation of autofluoerscent material in cortical neurons of Spatacsin KO mice (B). The neuron loss was paralleled by an activation of astrocytes in Spatacsin KO mice as evident from glial fibrillary acidic protein (GFAP) stainings (D). Cortical layers are labeled (I–VI). **(E-H)** Accumulation of autofluorescent material was also obvious in the cerebellum of Spatacsin KO mice (F). As in the cortex the neuron loss was paralleled by an activation of astrocytes in Spatacsin KO mice (H). GL: granular cell layer, PKC: Purkinje cell layer, ML: molecular layer. **(I-X)** Though neurons in the hippocampus (I-J), vestibular nuclei (VN) (M,O), inferior olivary nucleus (ION) (N,P) and in the spinal cord (U,V) accumulate autofluorescent material, no activation of astroglia was observed for these regions (K,L,Q-T,W,X). Scale bars: 150 (A-H), 250 (I-L,U-X), and 50 μm (M-T). CA1: Cornu ammonis 1. CA3: Cornu ammonis 3. DG: dentate gyrus. AF: autofluorescence.

Neuron loss in the cortex (Fig 4A and 4B) and the cerebellum (Fig 4E and 4F) was preceded by intraneuronal accumulation of autofluorescent material (emission wavelength 460–630 nm) and paralleled by the activation of astrocytes as evident from GFAP stainings (Fig 4C, 4D, 4G and 4H). Cells accumulating autofluorescence co-labeled with β-galactosidase, Ctip2, SatB2, parvalbumin or calbindin suggesting that principal cells as well as inhibitory interneurons are affected (S4 Fig). Though autofluorescent material also accumulated in other regions of the central nervous system like hippocampus (Fig 4I and 4J), different nuclei in the brain stem including the vestibular nuclei and the inferior olivary nucleus (Fig 4M–4P), and spinal cord neurons (Fig 4U and 4V), there was no evidence for astrocyte activation in these regions (Fig 4K, 4L, 4Q–4T, 4W and 4X).

### Degenerating neurons accumulate autofluorescent autolysosome-related material

**Fig 5:**
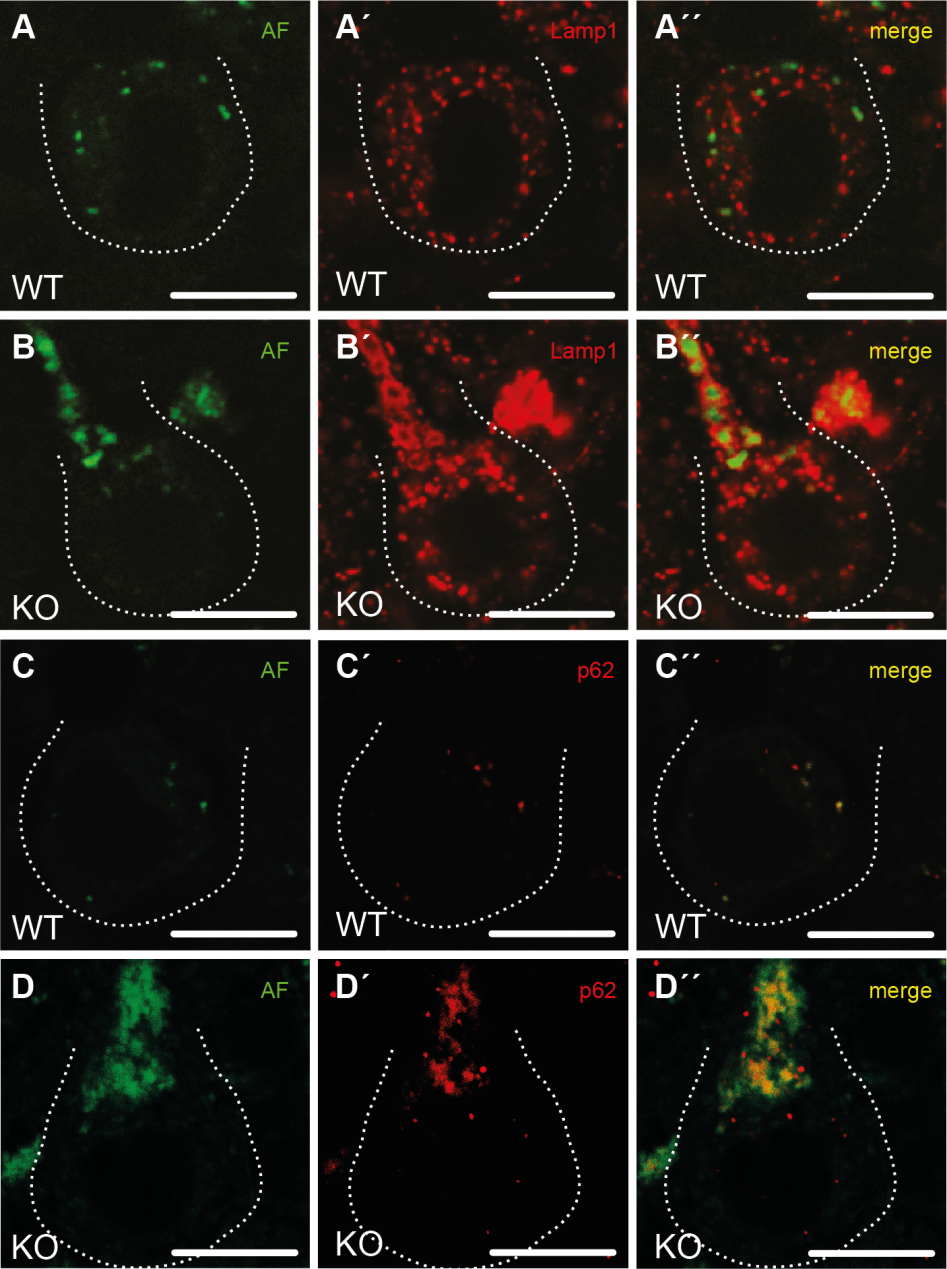
Purkinje cells in Spatacsin knockout mice accumulate abnormal autolysosomes. **(A-B”)** While the small autofluorescent particles (AF) in WT Purkinje cells (A”) are rarely associated with the lysosomal membrane protein Lamp1, autofluorescent structures in KO Purkinje cells stain for Lamp1 at their periphery (B”). **(C-D”)** The autofluorescent material in KO Purkinje cells contains p62 (D”) suggesting that the vesicular structures positive for both Lamp1 and p62 represent autolysosomes. Purkinje cell somata are marked by a dashed line. Age at sampling: 16 months. AF: autofluorescence; Scale bars: 10 μm.

To characterize the intraneuronal autofluorescent material in more detail, we stained brain sections for different subcellular marker proteins. Different from WT (Fig 5A–5A”), autofluorescent spots in Purkinje cells of KO mice were large, often clustered and were surrounded by membranes positive for the lysosomal marker protein Lamp1 (Fig 5B–5B”). As the contents of these vesicular structures stained for p62 (Fig 5D–5D”), a receptor for cargo destined to be degraded by autophagy, the autofluorescent deposits likely represent undegraded autolysosomal material.

### The regeneration of lysosomes from autolysosomes is impaired upon disruption of Spatacsin

**Fig 6:**
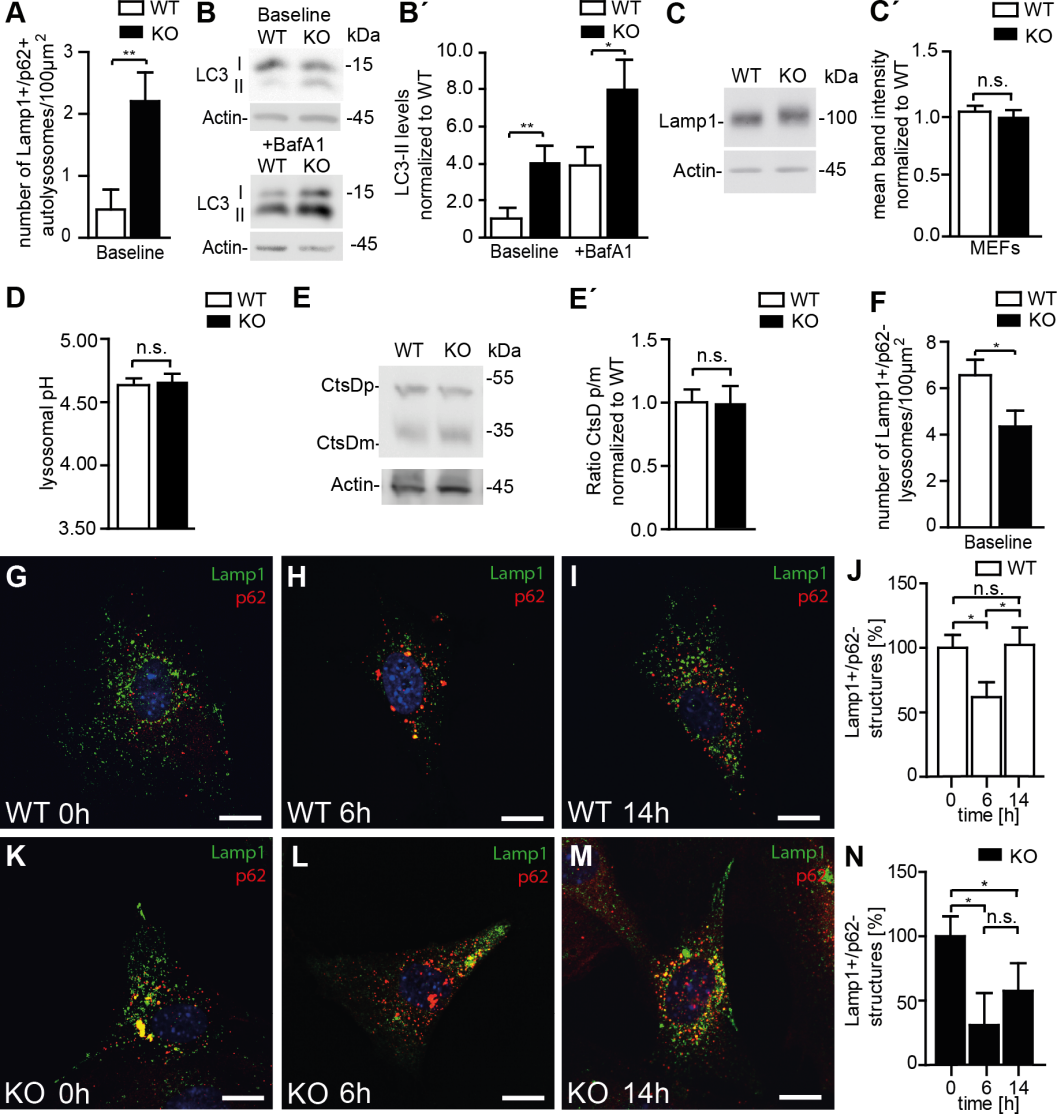
Disruption of Spatacsin decreases the regeneration of lysosomes in KO MEFs. **(A, G, K)** Autolysosomes defined as vesicles that stain for both Lamp1 and p62 are increased in KO compared to WT MEFs (Mean±SEM of n = 3 experiments; Student’s t-test: ** indicates p<0.01). **(B, B´)** LC3-II levels are increased in KO MEFs and further increase after treatment with bafilomycin A1. Quantification of LC3-II levels normalized to actin in MEFs (Mean±SEM of 4 independent experiments; Student´s t-test: ** indicates p<0.005 and * p<0.05). **(C, C´)** Overall Lamp1 levels are not changed in MEFs isolated from Spatacsin KO mice (Mean±SEM of n = 3 independent experiments; Student’s t-test: n.s. indicates not significant). **(D)** The intralysosomal pH is not changed in KO MEFs (Mean±SEM of n = 3 experiments with more than 1,000 lysosomes. Student’s t-test: n.s. indicated not significant). **(E, E´)** Lysosomal processing of the lysosomal protease Cathepsin D (CtsD) is not impaired in KO MEFs (CtsDp: precursor; CtsDm: mature). Mean±SEM of n = 4 independent experiments; Student’s t-test: not significant (n.s.). **(F)** Lysosomes defined as vesicles positive for Lamp1 but negative for p62 are decreased under baseline conditions in Spatacsin KO MEFs. (n = 3 experiments; Student’s t-test: * indicates p<0.05). **(G-N)** Upon 6 h of starvation lysosomes were depleted in both WT and KO MEFs. After 14 h lysosome numbers recovered to baseline levels only in WT (Mean±SEM of n = 3 experiments; Student’s t-test: * indicates p<0.05). Scale bars: 10μm.

Consistent with a defect of autolysosomal clearance, the number of autolysosomes, defined as vesicles positive for both Lamp1 and p62 (Fig 6A, 6G and 6K), and LC3-II levels (Fig 6B and 6B’) were increased in KO MEFs compared to WT. LC3-II levels further increased upon treatment with bafilomycin A1, which inhibits autolysosome acidification and hence autolysosomal degradation (Fig 6B and 6B’). Western analysis of Lamp1 levels did not reveal an alteration of overall Lamp1 levels in KO MEFs (Fig 6C and 6C’). Lysosomal pH as an important determinant for the activity of lysosomal proteases also did not differ between genotypes (Fig 6D) and the ratio between the mature and the precursor forms of the lysosomal protease cathepsin D was unchanged (Fig 6E and 6E’). The number of lysosomes, however, as defined as Lamp1-positive vesicular structures that did not co-stain for p62 was reduced in KO MEFs (Fig 6F). Though lysosomes were depleted upon induction of autophagy by starvation for 6 h in both WT and KO MEFs, only in WT lysosome numbers recovered to baseline after 14 h of ongoing starvation while they remained diminished in KO lysosomes (Fig 6G–6N and S5 Fig).

**Fig 7:**
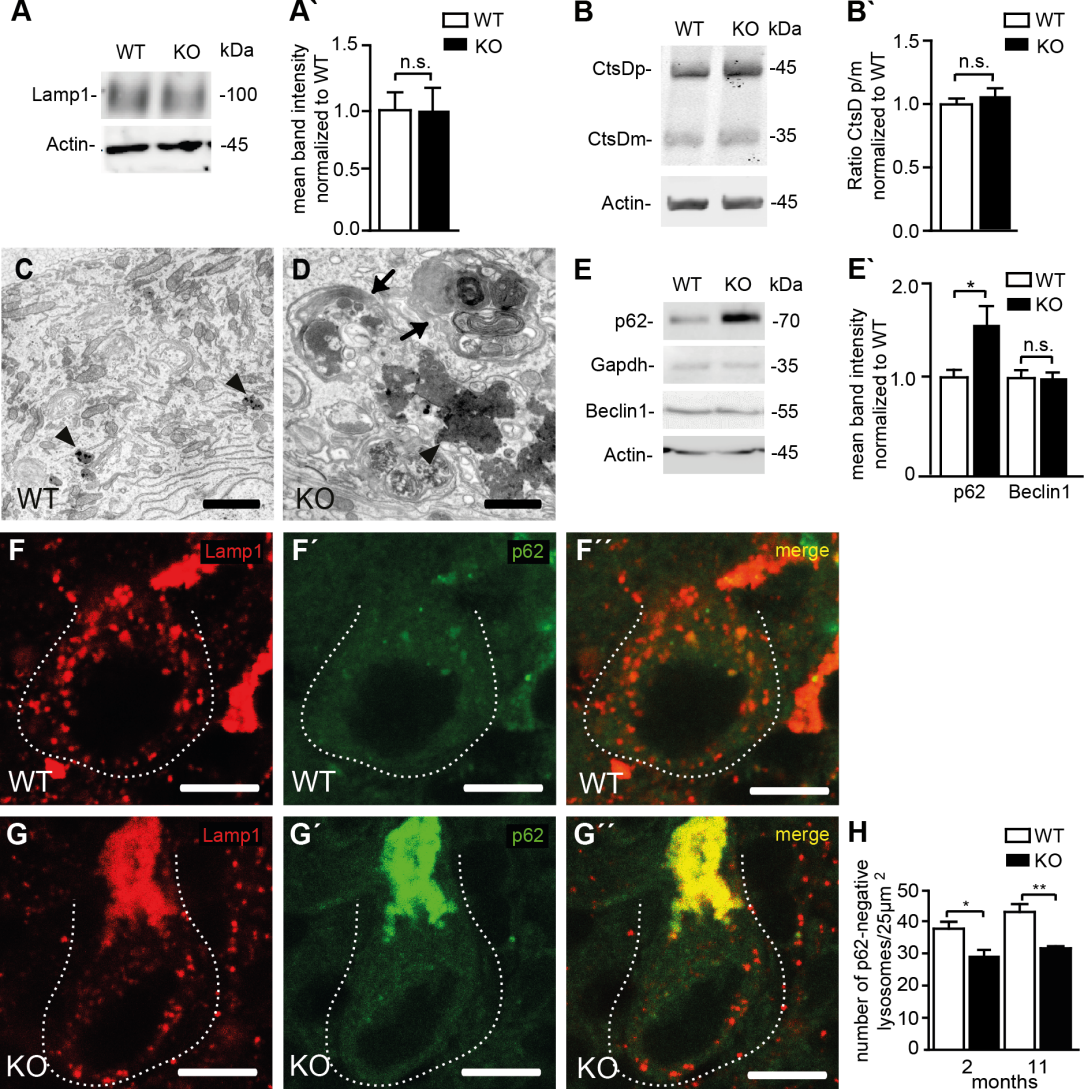
Reduced lysosome numbers in Purkinje cells of Spatacsin knockout mice are consistent with impaired autophagic lysosome reformation. **(A, A´)** Overall Lamp1 levels are not changed in Spatacsin KO brains (Mean±SEM of n = 3 experiments; Student’s t-test: n.s. indicates not significant). **(B, B´)** Lysosomal processing of the lysosomal protease Cathepsin D (CtsDp: precursor; CtsDm: mature) is not impaired in brain lysates of Spatacsin KO mice (Mean±SEM of n = 3 experiments; Student’s t-test: n.s. indicates not significant). **(C, D)** The ultrastructural analysis of KO Purkinje cells reveals clusters of vesicular structures filled with autophagic material (arrows) and irregularly shaped electron-dense lipofuscin-like deposits (D, arrowhead), while only some lipofuscin particles are found in WT (C, arrowheads). Scale bars: 2 μm. **(E, E´)** Levels of p62, a marker for cargo destined to be degraded by autophagy, are increased in Triton X-100 insoluble brain fractions of 16 months old Spatacsin KO mice, while Beclin 1, a key protein for the formation of autophagosomes, is not changed (Mean±SEM; n = 3 experiments; Student’s t-test: n.s. indicates not significant; * indicates p<0.05). **(F-H)** Lysosomes defined as vesicles positive for Lamp1 but negative for p62 are decreased in Purkinje cells of 2-month-old and 11-month-old (displayed in F-G´´) Spatacsin KO mice (Mean±SEM; n = 3 experiments; One-way ANOVA: * indicates p<0.05; ** indicates p<0.005). Scale bar: 10 μm.

We asked whether the results obtained in MEFs also apply to neurons. As in cultured MEFs, overall Lamp1 levels were unchanged (Fig 7A and 7A’) and the processing of cathepsin D was intact in KO samples as judged from Western blot analysis of brain lysates (Fig 7B and 7B’). Similar results were obtained for region specific lysates of cortex, hippocampus, cerebellum, and spinal cord (S6 Fig). As a correlate of impaired autolysosomal degradation the ultrastructural analysis of Purkinje cells revealed membrane-bound vesicles filled with heterogeneous material including organelle-like structures in Spatacsin KO but not in WT mice (Fig 7C and 7D). In KO samples we further observed an accumulation of electron-dense deposits of irregular shape reminiscent of lipofuscin interspersed between abnormal autolysosomes, while only some typical lipofuscin particles were found in controls of the same age (Fig 7C and 7D). Similar membranous bodies and lipofuscin-like material were also found in cortical, hippocampal, and spinal cord neurons of aged KO mice (S7 Fig). Levels of p62 were strongly elevated in the Triton X-100 insoluble fractions of KO whole brain lysates as well as in lysates of selected brain regions (S6 Fig), whereas levels of Beclin-1, one of the key proteins for the initial steps of autophagosome formation [20], were not changed (Fig 7E and 7E’). We next quantified Lamp1-positive and p62-negative vesicles in Purkinje cell somata of brain sections from 2-month-old and 11-month-old mice, respectively (Fig 7F–7H). Strikingly, the number of lysosomes was already decreased in Purkinje cells of Spatacsin KO mice at 2 months of age before we observed autofluorescent deposits or any signs of Purkinje cell degeneration. This alteration was preserved at 11 months of age. Similar results were also obtained for cortical motoneurons (S8 Fig).

## Discussion

To study the pathophysiology of SPG11 we generated a Spatacsin KO mouse model. Consistent with HSP and proving the assumption that SPG11 is caused by Spatacsin loss-of-function, axons of cortical motoneurons degenerate in Spatacsin KO mice. Similar to human patients affected by SPG11, KO mice developed a progressive spastic gait disorder with cerebellar ataxia during the course of the disease [21], while we did not observe a thinning of the corpus callosum, which is one of the main features of SPG11 patients [21]. This discrepancy may be explained by the fact that corpus callosum phenotypes strongly depend on the respective mouse strain [22,23].

Spatacsin KO mice progressively loose large diameter axons of the corticospinal tract similar to other mouse models for HSP. In contrast to mouse models for pure HSP [24–26] but similar to our findings for Zfyve26 [13] cortical motoneurons and Purkinje cells finally die. This neuron loss is paralleled by activation of astroglia as reported for other mouse models with neurodegeneration [27], while this is not the case in regions without overt neuron loss like hippocampus or spinal cord.

Since we did not observe structural changes or motor defects in KO mice younger than 12 months of age, disruption of Spatacsin does not entail obvious neurodevelopmental defects, as might have been expected from compromised axon outgrowth in neurons derived from induced pluripotent stem cells from SPG11 patients and upon siRNA-mediated Spatacsin knockdown in mouse cortical neurons [11].

Neuron loss in Spatacsin knockout mice was preceded by accumulation of intracellular autofluorescent material, which was associated with Lamp1-positive membranes. This is reminiscent of neuronal ceroid lipofuscinosis (NCL), lysosomal storage disorders characterized by lysosomal accumulation of autofluorescent ceroid lipopigments and neurodegeneration [28]. Because the autofluorescent deposits in Spatacsin KO mice also stained for p62, a receptor for material delivered into autophagosomes, these structures rather represent abnormal autolysosomes instead of lysosomes. Moreover, the ultrastructural analysis revealed membrane-bound vesicles filled with heterogeneous material including organelle-like structures at different stages of degradation consistent with autolysosomes in neurons from different regions. Along this line, membranous structures reported in sural nerve biopsies [29] and iPSC-derived neurons from SPG11 patients [11] may represent abnormal autolysosomes. Their accumulation indicates that autophagic clearance is impaired in Spatacsin KO, while the fusion of autophagosomes with lysosomes still occurs.

In agreement with our findings in Purkinje cells, the number of autolysosomes, characterized as vesicles labeled for both Lamp1 and p62, was increased in KO MEFs, which fits with previous results from siRNA-mediated knockdown of Spatacsin in HeLa cells [15]. Moreover, levels of LC3-II, the lipidated form of LC3 recruited to autophagosomal membranes, were increased as well. Since LC3-II levels in MEFs further increased upon treatment with bafilomycin A1, which inhibits lysosomal acidification and hence autolysosome clearance [30], autophagy does not appear to be completely blocked in Spatacsin deficient cells. Because fibroblast proliferation was unchanged in SPG11 patients [14], compromised autophagy upon disruption of Spatacsin may be less critical for fibroblasts than for postmitotic cells like neurons. Notably, it was reported that disruption of either Atg5 or Atg7 in neurons, which nearly abolishes autophagy completely, caused a loss of cortical neurons and Purkinje cells within the first 6 postnatal weeks while other types of neurons were less sensitive [31,32]. Thus the milder phenotype in Spatacsin KO mice, in which motoneurons and Purkinje cells are preserved at 2 months of age, is compatible with a partial impairment of autophagy. It appears that cortical motoneurons and Purkinje cells are particularly sensitive to autophagy defects. The long axonal projections of cortical motoneurons and the complex dendritic arbors of Purkinje cells may render these cells particularly sensitive for secondary transport defects because of accumulation of autophagy substrates. Indeed, axonal transport was compromised in neurons derived from induced pluripotent stem cells obtained from SPG11 patients and upon siRNA mediated Spatacsin knockdown in cortical mouse neurons [11].

The defect of autophagic clearance observed upon disruption of Spatacsin could arise from a primary lysosomal defect, as Spatacsin has been shown to interact with the adaptor protein complex AP-5, which was suggested to play a role for endosomal sorting [7,12]. Accordingly mistargeting of proteins normally destined for lysosomes or missorted cargo proteins may accumulate within lysosomes. Both situations may result in lysosomal dysfunction and hence a diminished turnover of autolysosomes as suggested for different lysosomal disorders [33–35]. Consistent with data obtained upon knockdown of Spatacsin in HeLa cells [15], a major lysosomal defect in Spatacsin KO cells is rather unlikely, because the processing of the lysosomal protease cathepsin D and the lysosomal pH were unchanged. Instead of a lysosomal defect we observed a depletion of lysosomes in both MEFs and Purkinje cells of KO mice. Lysosomes can either be generated through the endosomal pathway via the trans-Golgi network [36] or can be regenerated from autolysosomes via a process called autophagic lysosome reformation (ALR). The latter process is characterized by budding of “protolysosomal tubules” from autolysosomes, which finally separate and mature into functional lysosomes [16,37]. In HeLa cells depleted for either Spatacsin or Spastizin these tubules did not evolve upon serum starvation and hence it was proposed that impaired ALR may underlie SPG11 and SPG15 [15]. Our finding that in Spatacsin KO MEFs lysosomes are not recovered after prolonged starvation together with diminished lysosome numbers in cortical motoneurons and Purkinje cells support this conclusion for SPG11.

Taken together, our observations provide first evidence that ALR, which has so far only been observed *in vitro* [16,37], is also relevant *in vivo*. Along this line we propose that a reduction of lysosomes available for fusion with autophagosomes upon disruption of Spatacsin causes a defect in autolysosomal degradation, a consecutive accumulation of undegraded material and finally neuronal death.

## Materials and Methods

### Generation of Spatacsin knockout mice

To disrupt Spatacsin in mice, we used the EUCE0085_F05 embryonic stem cell clone E14 (EUCOMM) harbouring of a genetrap cassette in the first intron of the *Spg11* gene. This clone was injected into C57BL/6 donor blastocysts and transferred into foster mice. The resulting chimeric mice were mated with C57BL/6 mice to obtain heterozygous gene-trapped mice, which were subsequently mated to obtain homozygous trapped mice. For genotyping genomic DNA was isolated from tail biopsies. The primers “for” (cggctgcgggcagtctccaagtgc), “rev” (gggatgggaaaggttccgagaggc), and “cas_rev” (cgactcagtcaatcggaggactgg) were used in a single PCR reaction. The primer pair for/rev amplified a 256 bp fragment for the wild-type allele and the primer pair for/cas_rev a 167 bp fragment for the trapped allele. Experiments were performed on a mixed 129SvJ/C57BL/6 background in the 4^th^ to 6^th^ generation. Mice were housed in a 12 h light/dark cycle and fed on a regular diet *ad libitum*.

All animal experiments were approved by the Thüringer Landesamt für Lebensmittelsicherheit und Verbraucherschutz (TLLV) (application number 02-016/13) and were conducted under strict accordance with the ARRIVE guidelines.

### Behavioral analysis

Beam-walking and coordination test were performed on a horizontal plastic beam (1,000 mm long, 40 mm broad, 20 cm elevated from the ground) leading to the home cage as previously described [13]. For Rotarod analysis mice were placed on the rotating rod of the apparatus (Ugo basile). After constant speed (4 rpm) for a maximum of 2 min the speed was continuously accelerated (4–40 rpm in 5 min), and the latency until mice fell off the beam was recorded. The mean from two independent trials per day was used for statistical analysis.

### Northern blot analysis

For the probe we amplified a 602 bp cDNA fragment (part of exon 30 of *Spg11*) from mouse brain cDNA with the forward primer 5’-gcaaacactaacacacactccgcagtgg-3’ and the reverse primer 5’-gcaacaccagcactagatcctggc-3’. Northern blot analysis was performed as described previously [38].

### Antibodies

Monoclonal antibodies were raised against the epitope EKLSSGSISRDD (amino acids 1400–1411) of the Spatacsin protein in BALB/C mice (c346, Abmart). The affinity-purified antibody was used in a dilution of 1:50. Our polyclonal rabbit anti-Zfyve26 antibody described previously [13] was used at a dilution of 1:50.

The following commercially available antibodies were used: mouse anti-Calnexin (1:1,000, BD Biosciences); goat anti-Ap5b1 (1:500, Santa Cruz); rabbit anti-β-Galactosidase (1:250, Chemicon); rabbit anti-Calbindin D-28K (1:1,000, Millipore); mouse anti-parvalbumin (1:5,000, Swant); rat anti-Ctip2 (1:200, Abcam), mouse anti-SatB2 (1:100, Santa Cruz); α-bungarotoxin conjugated with Alexa Fluor555 (1:500, Life Technologies); mouse anti-NeuN (1:1,000, Millipore); mouse anti-GFAP (1:1,000, Millipore); rat anti-Lamp1 (1:500 for immunofluorescence studies; 1:1,000 for immunoblots, BD Pharmigen); rabbit anti-LC3 (1:500 for immunoblots, Novus Biologicals); mouse anti-p62 (1:250 used for immunofluorescence studies; 1:1,000 used for immunoblots, Abcam); rabbit anti-Beclin-1 (1:500, Santa Cruz); goat anti-CtsD (1:500, Santa Cruz); rabbit anti-β-actin (1:2000, Abcam); goat anti-Gapdh (1:500, Santa Cruz). Horseradish peroxidase—labelled secondary antibodies for Western blotting: goat anti-rabbit and goat anti-mouse (both 1:4,000, Amersham Bioscience); goat anti-rat (1:2,000, Santa Cruz); rabbit anti-goat (1:1,000, Sigma-Aldrich). Fluorescently labelled secondary antibodies: goat anti-rabbit, goat anti-mouse, or goat anti-rat coupled with Alexa 488 and Alexa 546, respectively (1:1,000, Life Technologies); goat anti-mouse, goat anti-rabbit or goat anti-rat coupled with Cy5 (1:1,000, Jackson ImmunoResearch Laboratories). Nuclei were counterstained with Hoechst-33258 (1:10,000; Molecular Probes).

### Protein lysates and immunoblotting

For immunoblotting brain tissue lysates were prepared as described [13]. Triton-X 100 insoluble fractions from total brain and spinal cord as well as from brain specific regions like cortex, hippocampus, and cerebellum were prepared from three mice per genotype as described previously [39]. For the hippocampus 6 hippocampi per genotype were pooled to prepare protein lysates. All samples were denatured for 5 minutes at 95°C in Laemmli buffer and separated by SDS PAGE and blotted onto PVDF membranes (Roche), which were blocked with 2.5% (w/v) milk powder and 2.5% (w/v) BSA in TBS-T (137 mM NaCl, 2.7 mM KCl, 19 mM Tris base, 1% (w/v) Tween). Proteins were either detected with the ECL Plus Western Blotting Detection System (GE Healthcare) on a LAS 4000 system (GE Healthcare) or based on fluorescence using a LI-COR Odyssey detection system.

### Immunohistochemistry and morphological analysis

Animals were anaesthetized with isoflurane (Actavis) and perfused transcardially with 4% PFA in 1xPBS. Brains were removed and post-fixed in 4% PFA overnight at 4°C.

LacZ stainings of tissue sections were performed as described [40].

For histological analysis tissues were either embedded in paraffin or in Tissue-Tek (Sakura). Sections from paraffin embedded tissues were 8μm and cryosections 20μm thick. For histological analysis sections were stained either with hematoxylin/eosin or cresyl violet acetate (Nissl) according to the manufacturers’ protocols (Sigma-Aldrich). Images were captured with an Olympus DP70 microscope and further analysed by *ImageJ*. Pyramidal neurons in the *Stratum pyramidale* of the hippocampus were counted for corresponding regions and normalized to the respective area. The quantification of alpha-motoneurons and large diameter corticospinal axons was performed on semi-thin cervical and lumbar sections stained with Richard’s Blue [41]. Large diameter axons defined by a diameter > 4μm were counted. Alpha-motoneurons were identified because of the location in the ventral horn and their characteristic morphological appearance. Neuromuscular junctions were stained with α-bungarotoxin according to the manufacturer’s protocol (Life Technologies) in 20μm cryosections of either the gastrocnemius muscle from the hindlimb or the triceps brachii muscle from the forelimb, respectively. For quantification of cortical neurons 40μm free floating sagittal brain cryosections were stained for NeuN and mounted. Images of the motor cortex were taken with a Leica TCS SP5 confocal scanning fluorescence microscope. Neurons were quantified with the cell counter plug in and the area measurement tool of *ImageJ*.

Free floating 20μm sections of the brain or primary cells were rinsed three times with 1xPBS, then fixed for 15 minutes in 4% PFA in 1xPBS at room temperature and washed three times for 10 min in 1xPBS. 0.25% Triton-X in 1xPBS was used to permeabilize the cells. After rinsing the cells once with 1xPBS, blocking solution (5% goat serum in 1xPBS) was added. Primary and secondary antibodies were applied in blocking solution. Images were taken with a Leica TCS SP5 confocal scanning fluorescence microscope with the Z-stack module.

To analyze whether the autofluorescent deposits co-localize with subcellular markers in brain tissue sections the fluorescent signal of deposits and the Cy5 secondary antibodies were recorded and further analyzed by linear unmixing as described previously [13].

As lysosomes we defined Lamp1-positive but p62-negative vesicular structures, while autolysosomes are characterized by the presence of Lamp1 and p62. In order to analyze the number of lysosomes 40 μm thick sagittal brain sections were co-stained for Lamp1 and p62. Only sections of somata of Purkinje cells not extending beyond the image boundary and hit vertically in respect to the nucleus were selected. The images were recorded with the Leica TCS SP5 confocal scanning fluorescence microscope. The number of free lysosomes were counted and normalized to the area of the cell soma with *ImageJ*. Co-localization between Lamp1 and p62 as well as between fusion proteins and subcellular markers were performed in *BioImageXD* as described [42].

### Ultrastructural analysis

For semi- and ultrathin sections, 2 animals per genotype were perfused with 50 ml fixative (4% paraformaldehyde, 1% glutaraldehyde). Brain and spinal cord were removed and post-fixed overnight at 4°C. 150μm sagittal and coronal sections of brain and spinal cord were cut with a vibratome (Leica Microsystems) and processed as described [41]. Semithin sections were stained with Richard’s blue. Ultrathin 80 nm sections (Ultratome III, LKB Instruments) were mounted on filmed copper grids (100 mesh), post-stained with lead citrate, and studied in a transmission electron microscope (EM 900, Zeiss) at 80 kV.

### Primary cell culture

Mouse embryonic fibroblasts (MEFs) were prepared from E13.5 mouse embryos as described [13]. In order to assess the number of free lysosomes MEFs were cultured on 13 mm diameter coverslips (Marienfeld) in 24-well plate (Greiner) and maintained in DMEM medium (Life Technologies) with or without (starvation condition) 10% FBS and 2mM L-glutamine as described [37]. Cells at baseline conditions and cells starved for 6 and 14 h were fixed with 4% PFA in 1xPBS, rinsed with 1xPBS, and co-stained with anti-rat-Lamp1 and anti-mouse-p62 as described above. Images were digitally acquired by a Leica TCS SP5 confocal scanning fluorescence microscope and the number of Lamp1-positive and p62-negative lysosomes quantified with ImageJ. To inhibit autophagy cells were incubated with medium containing 100 nM Bafilomycin A1 (Santa Cruz) for 16h.

### Intralysosomal pH measurement in MEFs

Lysosomal pH measurements were carried out as described previously in [43]. More than 1,000 lysosomes from 3 independent experiments were analysed per genotype.

### Statistics

For repeated experiments two-way ANOVA followed by Bonferroni post-hoc tests were used to compare between genotypes. For morphological and quantitative western blot analysis Student’s two-tailed t-test was used. Data are shown as mean±SEM if not indicated otherwise.

## Supporting Information

S1 Fig Expression of *Spg11* in different brain regions of 8-month-old homozygous knockout mice. (**A**) The broad neuronal marker NeuN (green) and ß-Gal (red) largely overlap in the motorcortex. Scale bar: 200 μm. (**B**) Ctip2-positive cortical neurons also express *Spg11*. Scale bar: 200 μm. (**C**) In CA3 NeuN-positive neurons also express *Spg11*. Scale bar: 100 μm. (**D**) Some parvalbumin-positive interneurons in CA3 express *Spg11*. Scale bar: 100 μm. (**E**) Cerebellar granule neurons and Purkinje cells express *Spg11*. Scale bar: 25 μm. (**F**) Parvalbumin-positive interneurons in the cerebellum express *Spg11*. Scale bar: 25 μm. (**G, I**) NeuN and ß-Gal widely overlap in neurons of vestibular nuclei (G) and the inferior olivary nucleus (I). Scale bars: 100 μm. (**H, J**) Parvalbumin-positive interneurons also express *Spg11* in vestibular nuclei (H) and the inferior olivary nucleus (J). Scale bars: 100 μm.
(TIF)

S2 Fig The basic structure of motor endplates is not changed in Spatacsin knockout mice. **(A-D)** Visualization of α-bungarotoxin stained neuromuscular junctions of the gastrocnemius muscle from the hindlimb (A,B) and the triceps brachii muscle from the forelimb (C,D). Scale bars: 25 μm.
(TIF)

S3 Fig The number of hippocampal pyramidal neurons and spinal cord motoneurons is not reduced in 16-month-old Spatacsin knockout mice. (**A, B**) Paraffin sections of the CA1 region of the hippocampus of 16-month-old WT (A) and KO (B) mice. (**C**) Quantification of CA1 pyramidal neurons (n = 3; Student’s t-test: n.s. not significant). (**D, E**) Paraffin sections of the CA3 region of the hippocampus of 16-month-old WT (D) and KO (E) mice. (**F**) Quantification of CA3 pyramidal neurons (n = 3; Student’s t-test: n.s.: not significant). Scale bars: 25 μm (A, B, D, E). (**G, H**) Semithin cervical spinal cord sections. The border of alpha-motoneurons is indicated by a dashed line. (**I**) Quantification of cervical alpha-motoneurons (n = 3; Student’s t-test: n.s. not significant). (**J, K**) Semithin lumbar spinal cord sections. The border of alpha-motoneurons is indicated by a dashed line. (**L**) Quantification of lumbar alpha-motoneurons (n = 3; Student’s t-test: n.s. not significant). Scale bars: 25 μm (G, H, J, K).
(TIF)

S4 Fig Characterization of cells accumulating autofluorescent material in different brain regions of 8-month-old Spatacsin knockout mice. (**A, E, G, I, K, M**) Autofluorescent material accumulates in *Spg11* expressing cells in different regions of the central nervous system including cortex (A), hippocampus (E), cerebellum (G), vestibular nuclei (VN) and the inferior olivary nucleus (ION) in the brain stem (I,K) and the spinal cord (M). Scale bars: 150μm (A), 100μm (E), 25μm (G), 100μm (I, K), 50μm (M). (**C**) Autofluorescent material is also observed in SatB2-positive cortical neurons. Scale bar: 150μm. (**B, F, H, J, L**) Parvalbumin-positive interneurons also accumulate autofluorescent material in different regions of the central nervous system including cortex (B), hippocampus (F), cerebellum (H), and brain stem (J, L). Scale bars: 150 μm (B), 100 μm (F), 25 μm (H), 100 μm (J,L), 50 μm (N). (**D**) Autofluorescent material also accumulates in Ctip2-positive neurons in layer V of the cortex. Scale bar: 150 μm. (**N**) Autofluorescent material is present in calbindin-positive interneurons of the spinal cord. Scale bar: 50 μm. (O, O’) Alpha-motoneurons in the ventral horn of the spinal cord accumulate autofluorescent material as well in Spatacsin KO mice. Scale bar: 25 μm.
(TIF)

S5 Fig Single channels of stainings presented in Fig 6. **(A-A´´, D-D´´)** Under baseline conditions lysosomes determined as vesicles positive for Lamp1 but negative for p62 are decreased in Spatacsin KO MEFs. **(B-B´´, E-E´´)** After 6 h of starvation lysosomes were depleted in both WT and KO MEFs. (C-C´´, **F-F´´**) Following 14 h starvation the number of lysosomes only recovered to baseline levels in WT MEFs. Scale bars: 10 μm.
(TIF)

S6 Fig Quantification of Lamp1, Cathepsin D processing, and p62 in different brain regions. (**A-D**) Lamp1, p62, and the ratio between precursor Cathepsin D (CtsDp) and mature Cathepsin D (CtsDm) for cortex (A), hippocampus (B), cerebellum (C), and spinal cord (D) (n = 3; Student’s t-test: * indicates p<0.05; ** p<0.01; n.s. not significant). Because hippocampi had to be pooled because of the limited amount of material, a statistical analysis was precluded.
(TIF)

S7 Fig Autolysosomes and lipofuscin-like material in different brain regions. (**A-C**) Compared to WT the ultrastructural analysis of cortical neurons in 16-month-old KO mice reveals large clusters of irregularly shaped electron-dense lipofuscin-like deposits (B, arrowheads) and membranous structures filled with autophagic material (C, arrows). (**D, E**) In hippocampal pyramidal neurons of 16-month-old KO (E) mice the accumulation of lipofuscin-like material is quite prominent compared to WT (D). (**F**) Axonal swelling filled with autophagic vesicles in the corticospinal tract of 16-month-old KO mouse. Scale bars: 2 μm (A, B, D, E), 0.5 μm (C,F).
(TIF)

S8 Fig Reduced number of lysosomes in cortical motoneurons of Spatacsin knockout mice. (**A-C**) Lysosomes defined as vesicles positive for Lamp1 but negative for p62 are decreased in cortical motoneurons of 16-month-old Spatacsin KO (A-A”) compared to WT (B-B”) mice (Mean±SEM; n = 22 cells each; One-way ANOVA: * indicates p<0.05; ** indicates p<0.005). The border of cortical motoneurons is indicated by a dashed line. Scale bars: 10 μm.
(TIF)
