# Supplementary figures and images for: In Vivo Evidence for Lysosome Depletion and Impaired Autophagic Clearance in Hereditary Spastic Paraplegia Type SPG11
Source: PLoS Genet. 2015 Aug 18;11(8):e1005454. doi: 10.1371/journal.pgen.1005454 (PMC4540459; doi:10.1371/journal.pgen.1005454)

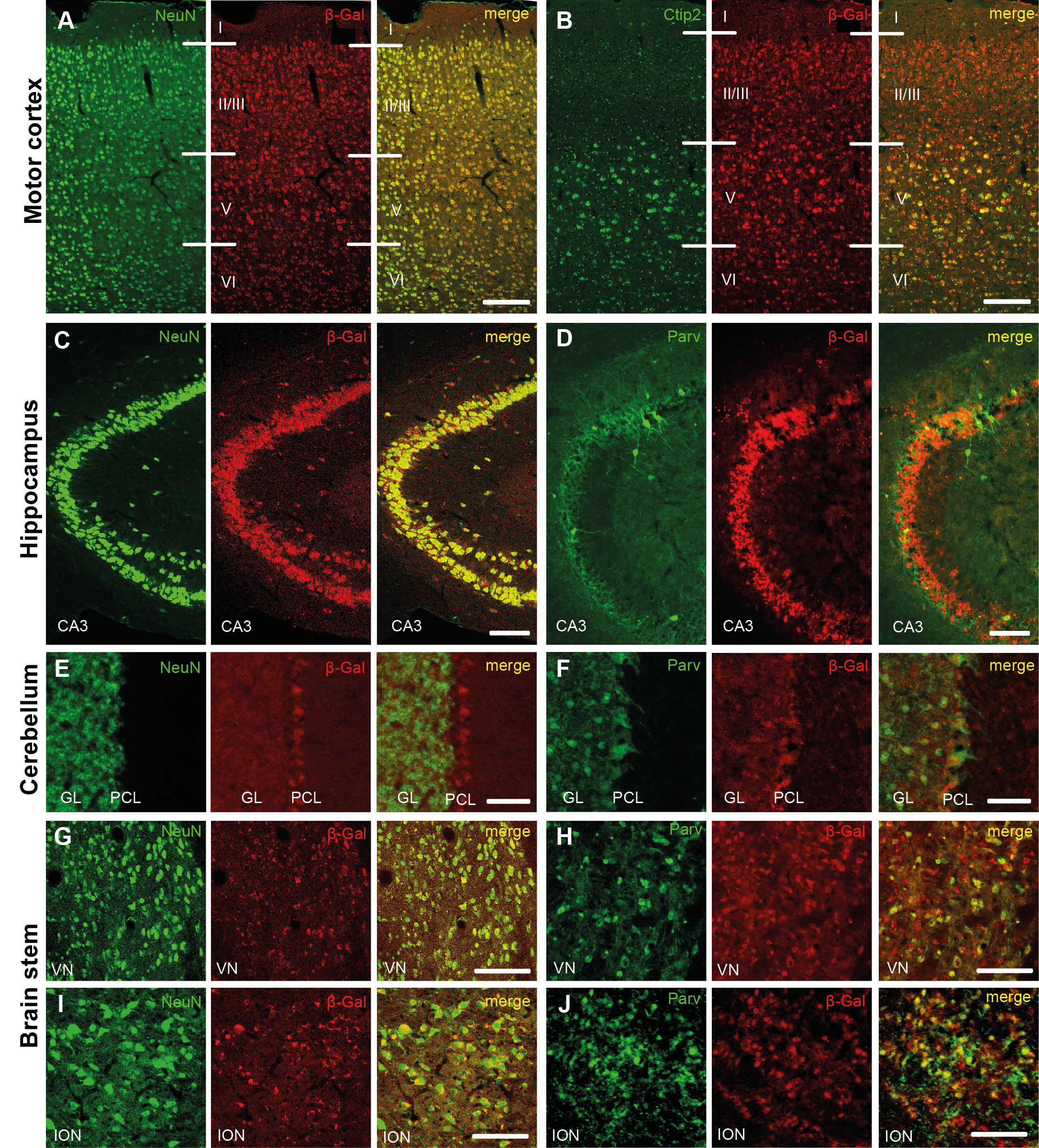

Supplement: S1 Fig — (A) The broad neuronal marker NeuN (green) and ß-Gal (red) largely overlap in the motorcortex. Scale bar: 200 μm. (B) Ctip2-positive cortical neurons also express Spg11. Scale bar: 200 μm. (C) In CA3 NeuN-positive neurons also express Spg11. Scale bar: 100 μm. (D) Some parvalbumin-positive interneurons in CA3 express Spg11. Scale bar: 100 μm. (E) Cerebellar granule neurons and Purkinje cells express Spg11. Scale bar: 25 μm. (F) Parvalbumin-positive interneurons in the cerebellum express Spg11. Scale bar: 25 μm. (G, I) NeuN and ß-Gal widely overlap in neurons of vestibular nuclei (G) and the inferior olivary nucleus (I). Scale bars: 100 μm. (H, J) Parvalbumin-positive interneurons also express Spg11 in vestibular nuclei (H) and the inferior olivary nucleus (J). Scale bars: 100 μm. (TIF) [file pgen.1005454.s001.tif]

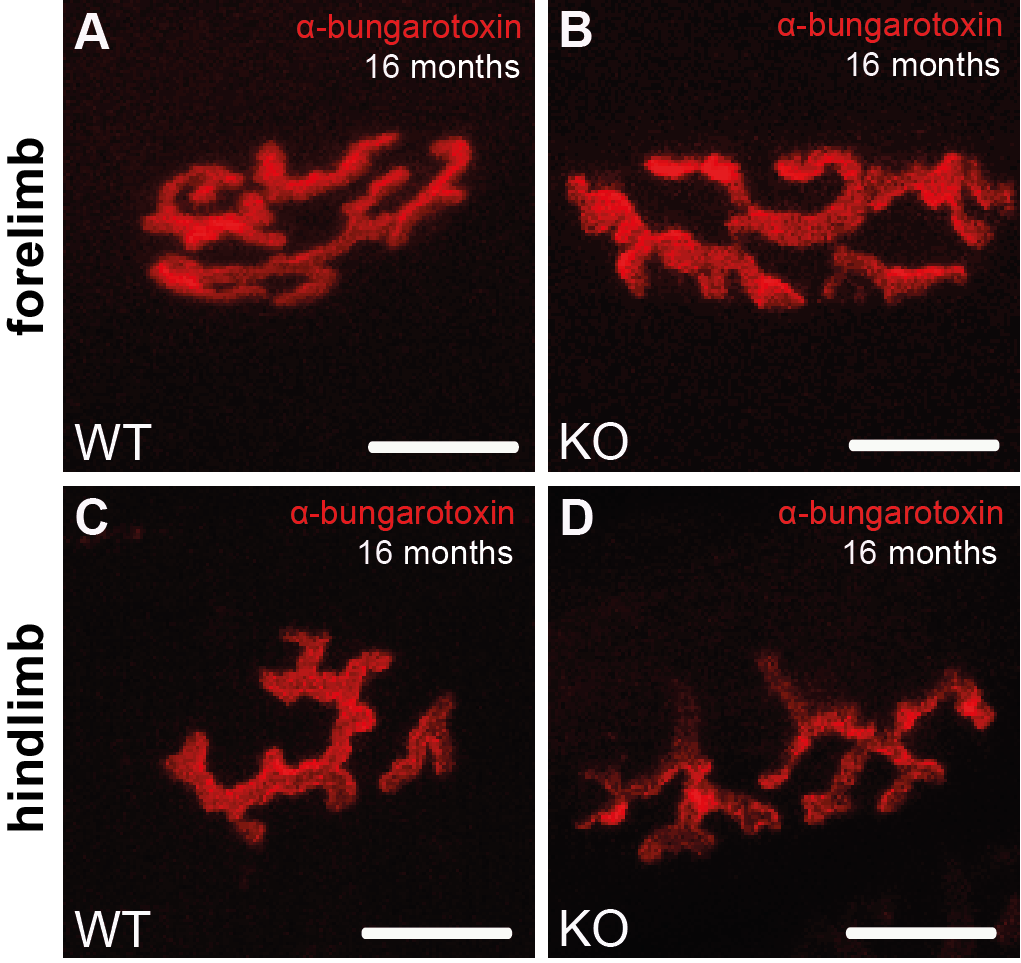

Supplement: S2 Fig — (A-D) Visualization of α-bungarotoxin stained neuromuscular junctions of the gastrocnemius muscle from the hindlimb (A,B) and the triceps brachii muscle from the forelimb (C,D). Scale bars: 25 μm. (TIF) [file pgen.1005454.s002.tif]

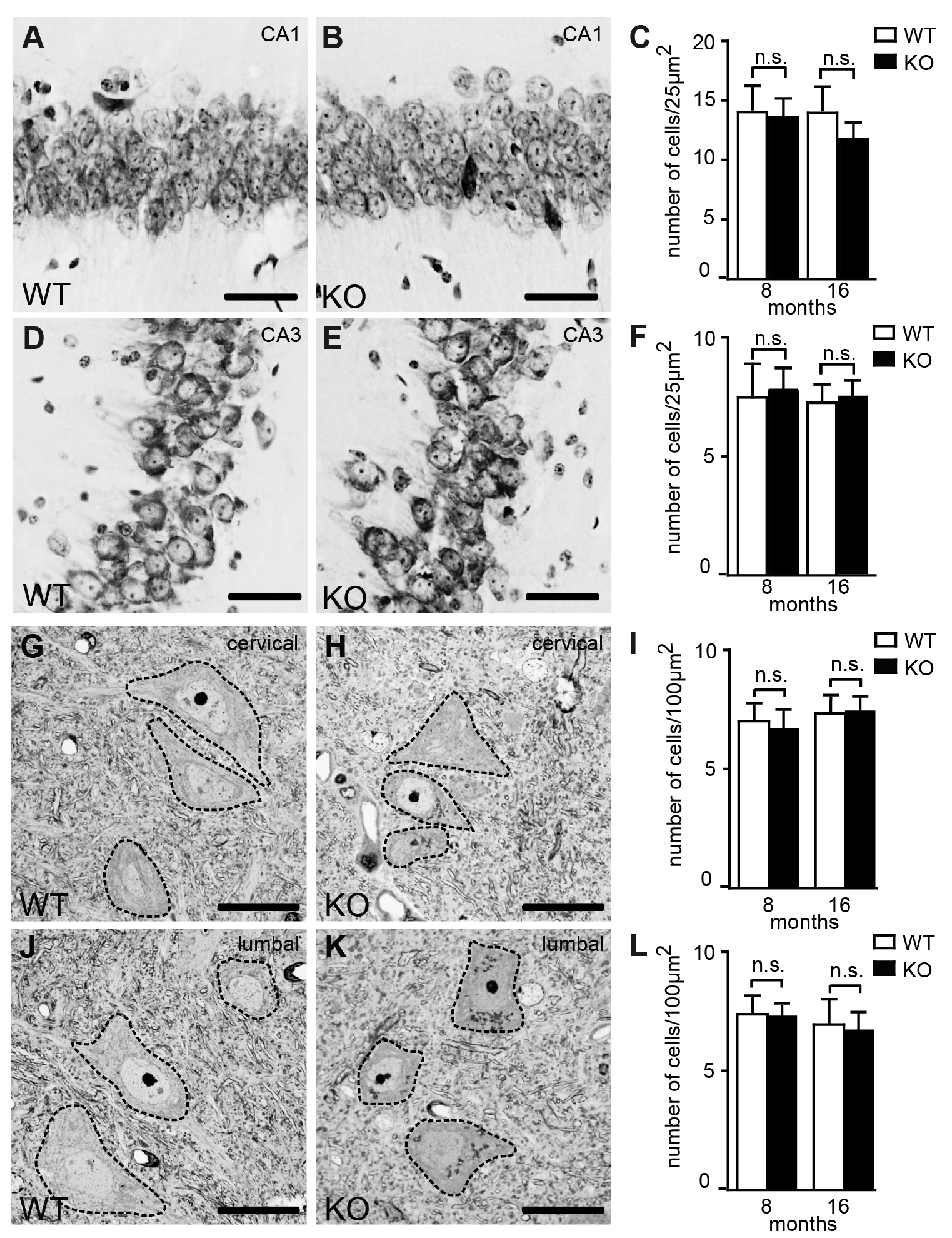

Supplement: S3 Fig — (A, B) Paraffin sections of the CA1 region of the hippocampus of 16-month-old WT (A) and KO (B) mice. (C) Quantification of CA1 pyramidal neurons (n = 3; Student’s t-test: n.s. not significant). (D, E) Paraffin sections of the CA3 region of the hippocampus of 16-month-old WT (D) and KO (E) mice. (F) Quantification of CA3 pyramidal neurons (n = 3; Student’s t-test: n.s.: not significant). Scale bars: 25 μm (A, B, D, E). (G, H) Semithin cervical spinal cord sections. The border of alpha-motoneurons is indicated by a dashed line. (I) Quantification of cervical alpha-motoneurons (n = 3; Student’s t-test: n.s. not significant). (J, K) Semithin lumbar spinal cord sections. The border of alpha-motoneurons is indicated by a dashed line. (L) Quantification of lumbar alpha-motoneurons (n = 3; Student’s t-test: n.s. not significant). Scale bars: 25 μm (G, H, J, K). (TIF) [file pgen.1005454.s003.tif]

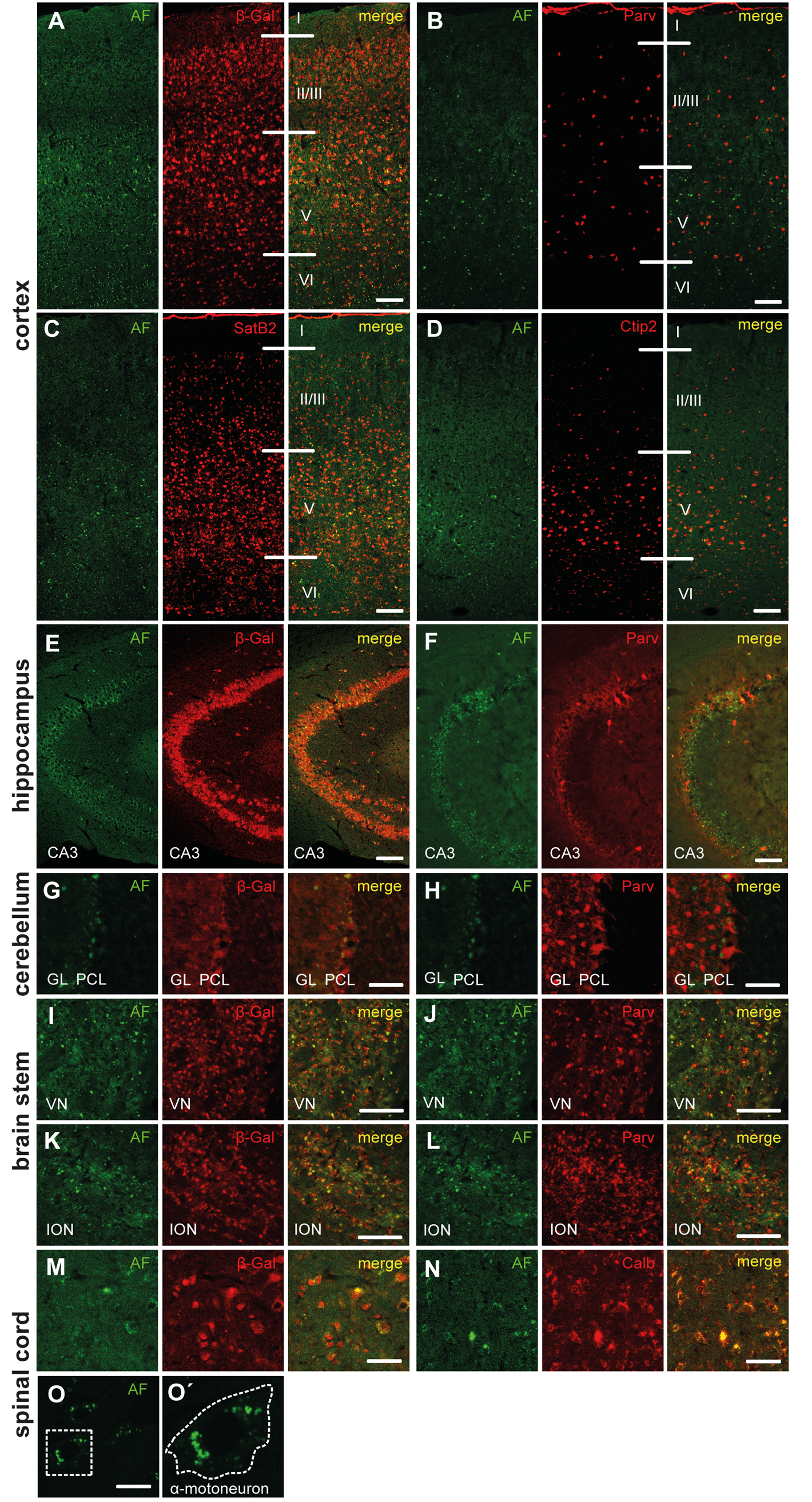

Supplement: S4 Fig — (A, E, G, I, K, M) Autofluorescent material accumulates in Spg11 expressing cells in different regions of the central nervous system including cortex (A), hippocampus (E), cerebellum (G), vestibular nuclei (VN) and the inferior olivary nucleus (ION) in the brain stem (I,K) and the spinal cord (M). Scale bars: 150μm (A), 100μm (E), 25μm (G), 100μm (I, K), 50μm (M). (C) Autofluorescent material is also observed in SatB2-positive cortical neurons. Scale bar: 150μm. (B, F, H, J, L) Parvalbumin-positive interneurons also accumulate autofluorescent material in different regions of the central nervous system including cortex (B), hippocampus (F), cerebellum (H), and brain stem (J, L). Scale bars: 150 μm (B), 100 μm (F), 25 μm (H), 100 μm (J,L), 50 μm (N). (D) Autofluorescent material also accumulates in Ctip2-positive neurons in layer V of the cortex. Scale bar: 150 μm. (N) Autofluorescent material is present in calbindin-positive interneurons of the spinal cord. Scale bar: 50 μm. (O, O’) Alpha-motoneurons in the ventral horn of the spinal cord accumulate autofluorescent material as well in Spatacsin KO mice. Scale bar: 25 μm. (TIF) [file pgen.1005454.s004.tif]

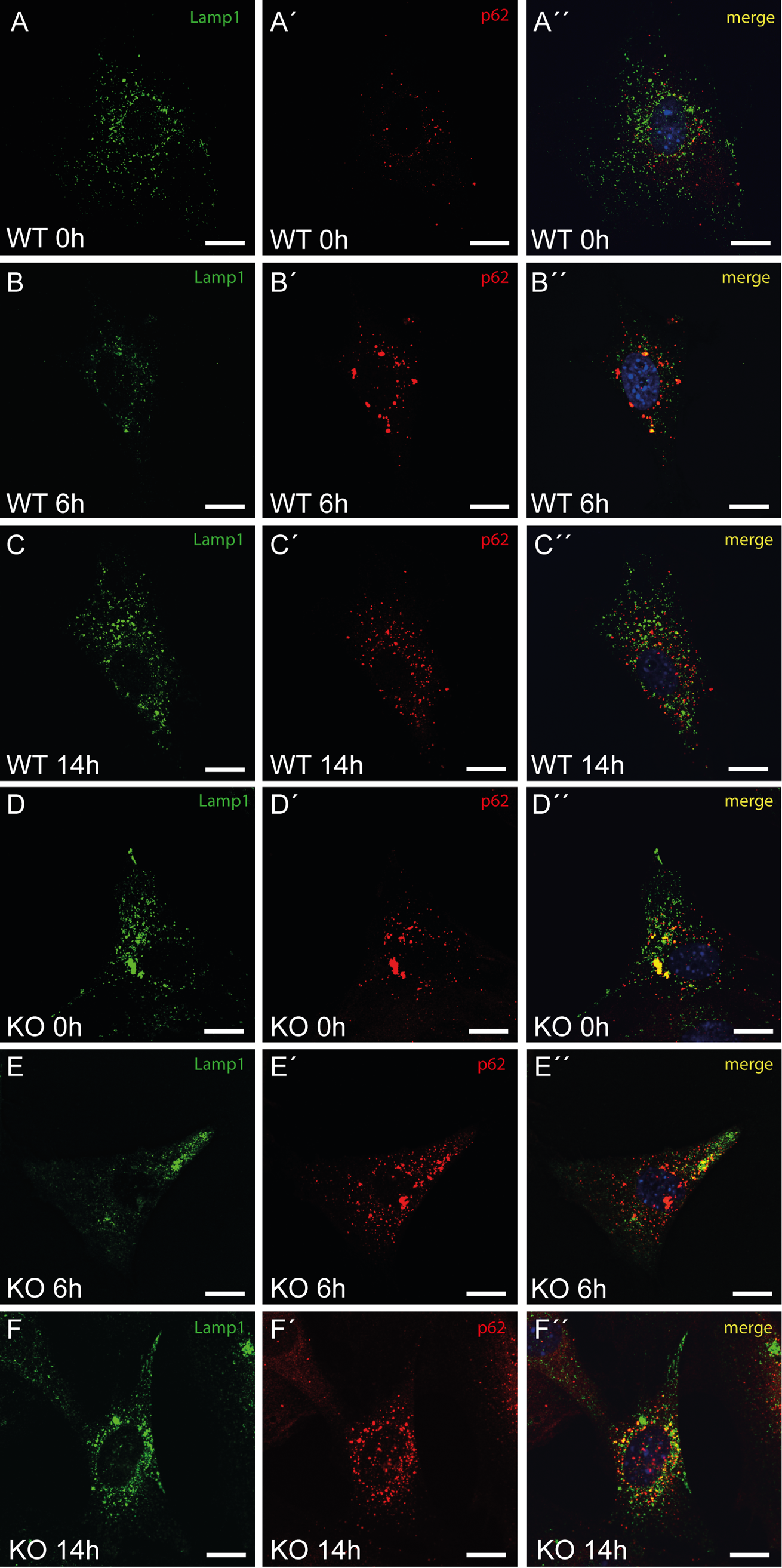

Supplement: S5 Fig — (A-A´´, D-D´´) Under baseline conditions lysosomes determined as vesicles positive for Lamp1 but negative for p62 are decreased in Spatacsin KO MEFs. (B-B´´, E-E´´) After 6 h of starvation lysosomes were depleted in both WT and KO MEFs. (C-C´´, F-F´´) Following 14 h starvation the number of lysosomes only recovered to baseline levels in WT MEFs. Scale bars: 10 μm. (TIF) [file pgen.1005454.s005.tif]

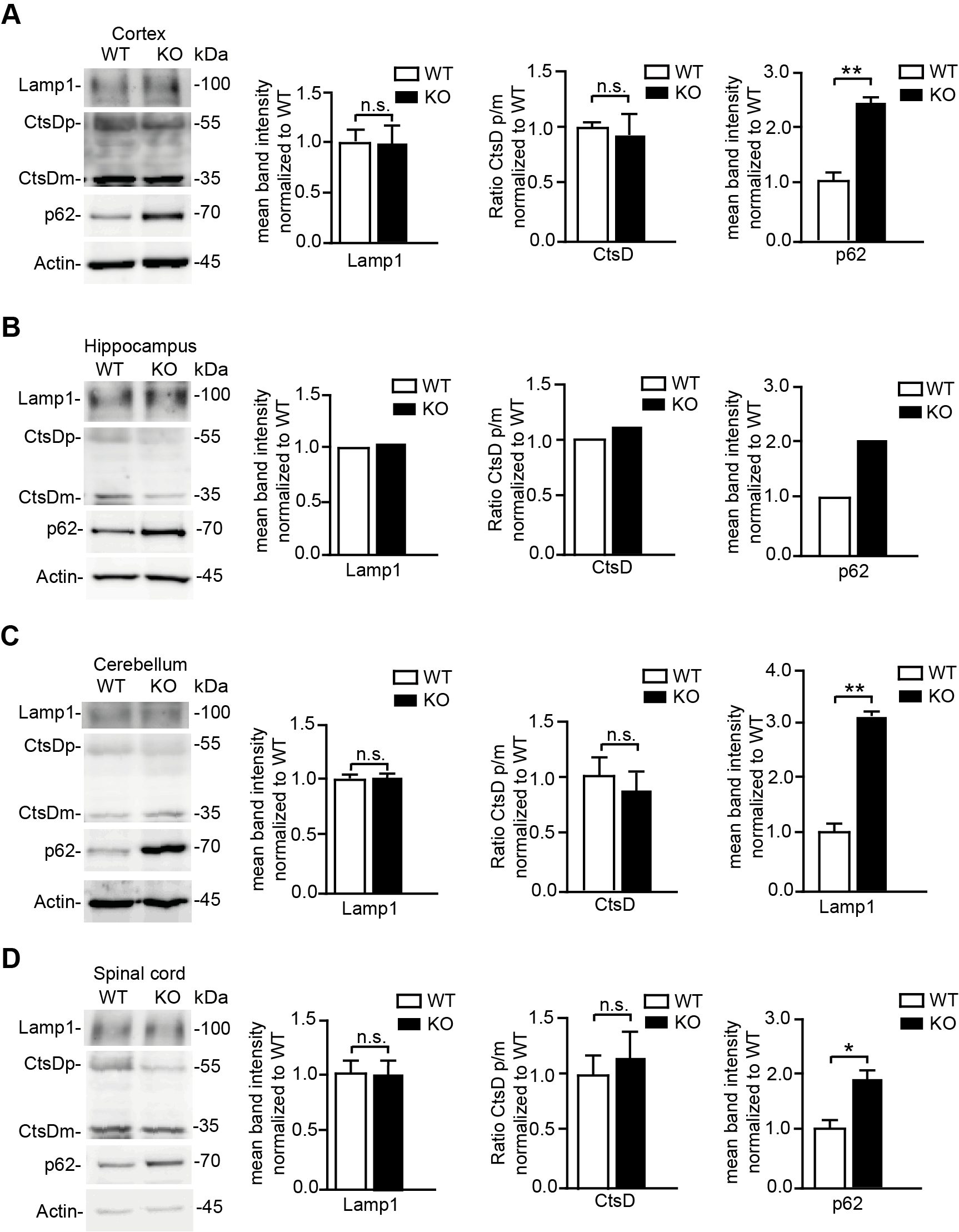

Supplement: S6 Fig — (A-D) Lamp1, p62, and the ratio between precursor Cathepsin D (CtsDp) and mature Cathepsin D (CtsDm) for cortex (A), hippocampus (B), cerebellum (C), and spinal cord (D) (n = 3; Student’s t-test: * indicates p<0.05; ** p<0.01; n.s. not significant). Because hippocampi had to be pooled because of the limited amount of material, a statistical analysis was precluded. (TIF) [file pgen.1005454.s006.tif]

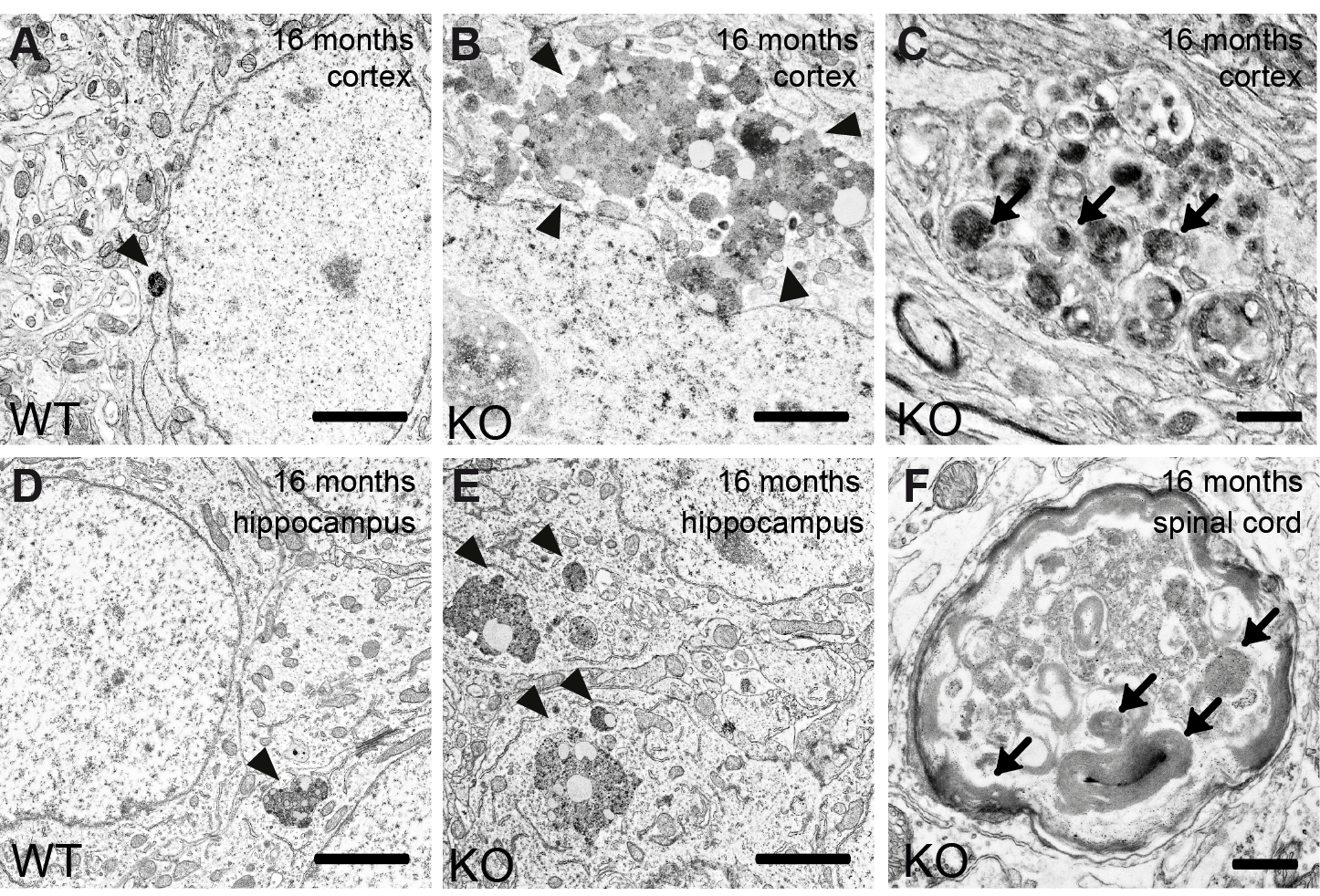

Supplement: S7 Fig — (A-C) Compared to WT the ultrastructural analysis of cortical neurons in 16-month-old KO mice reveals large clusters of irregularly shaped electron-dense lipofuscin-like deposits (B, arrowheads) and membranous structures filled with autophagic material (C, arrows). (D, E) In hippocampal pyramidal neurons of 16-month-old KO (E) mice the accumulation of lipofuscin-like material is quite prominent compared to WT (D). (F) Axonal swelling filled with autophagic vesicles in the corticospinal tract of 16-month-old KO mouse. Scale bars: 2 μm (A, B, D, E), 0.5 μm (C,F). (TIF) [file pgen.1005454.s007.tif]

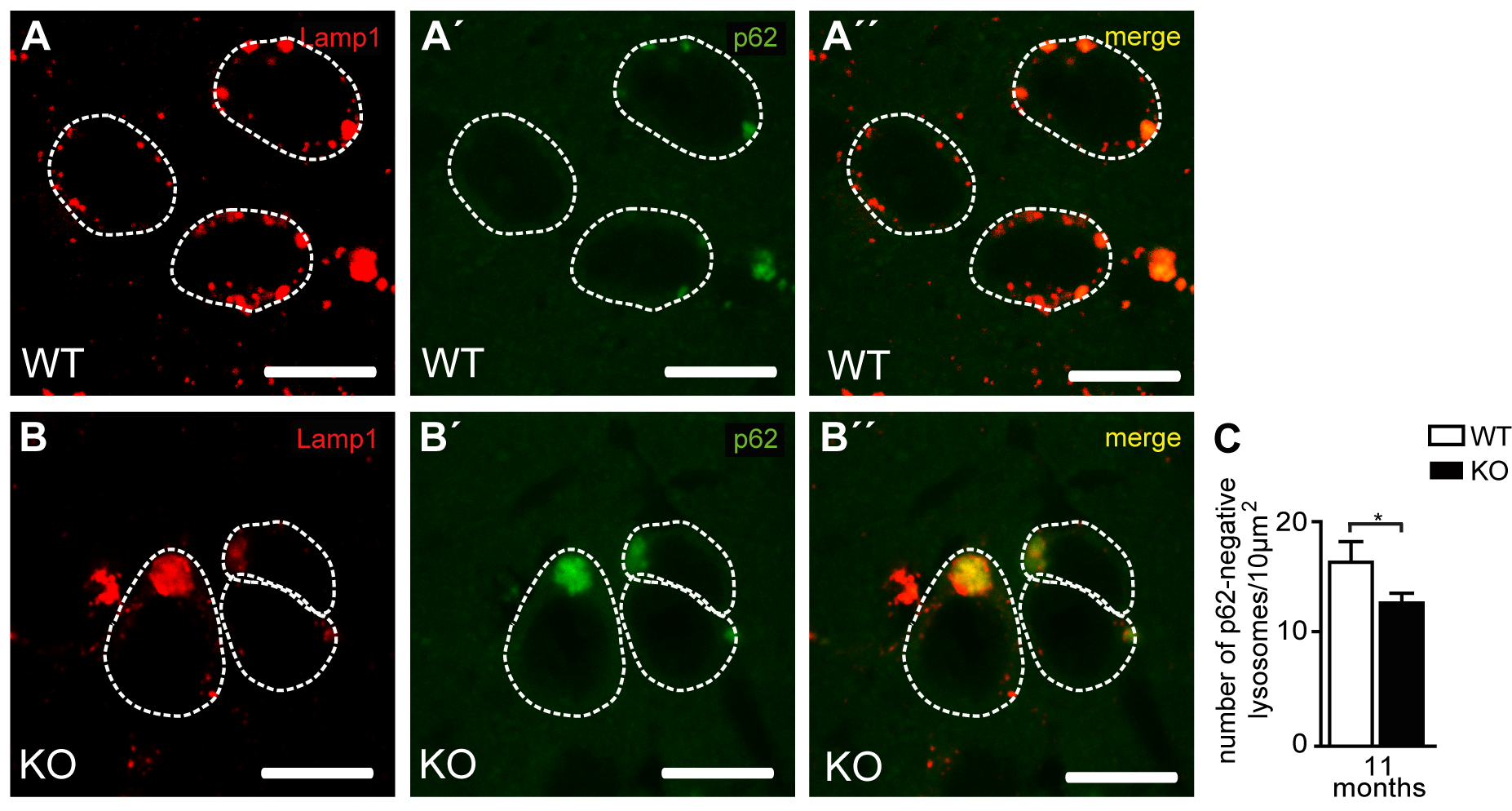

Supplement: S8 Fig — (A-C) Lysosomes defined as vesicles positive for Lamp1 but negative for p62 are decreased in cortical motoneurons of 16-month-old Spatacsin KO (A-A”) compared to WT (B-B”) mice (Mean±SEM; n = 22 cells each; One-way ANOVA: * indicates p<0.05; ** indicates p<0.005). The border of cortical motoneurons is indicated by a dashed line. Scale bars: 10 μm. (TIF) [file pgen.1005454.s008.tif]
